# Effectiveness of an Individualized Exergame-Based Motor-Cognitive Training Concept Targeted to Improve Cognitive Functioning in Older Adults With Mild Neurocognitive Disorder: Study Protocol for a Randomized Controlled Trial

**DOI:** 10.2196/41173

**Published:** 2023-02-06

**Authors:** Patrick Manser, Lars Michels, André Schmidt, Filip Barinka, Eling D de Bruin

**Affiliations:** 1 Motor Control and Learning Group – Institute of Human Movement Sciences and Sport Department of Health Sciences and Technology ETH Zurich Zurich Switzerland; 2 Department of Neuroradiology University Hospital Zurich Zurich Switzerland; 3 Department of Psychiatry University of Basel Basel Switzerland; 4 Clinic for Neurology Hirslanden Hospital Zurich Zurich Switzerland; 5 Department of Neurobiology, Care Sciences and Society Karolinska Institute Stockholm Sweden; 6 Department of Health OST – Eastern Swiss University of Applied Sciences St. Gallen Switzerland

**Keywords:** cognition, cognitive impairment, effectiveness, exercise, exergame, neuroplasticity, neurosciences, technology, training

## Abstract

**Background:**

Simultaneous motor-cognitive training is considered promising for preventing the decline in cognitive functioning in older adults with mild neurocognitive disorder (mNCD) and can be highly motivating when applied in the form of exergaming. The literature points to opportunities for improvement in the application of exergames in individuals with mNCD by developing novel exergames and exergame-based training concepts that are specifically tailored to patients with mNCD and ensuring the implementation of effective training components.

**Objective:**

This study systematically explores the effectiveness of a newly developed exergame-based motor-cognitive training concept (called “Brain-IT”) targeted to improve cognitive functioning in older adults with mNCD.

**Methods:**

A 2-arm, parallel-group, single-blinded randomized controlled trial with a 1:1 allocation ratio (ie, intervention: control), including 34 to 40 older adults with mNCD will be conducted between May 2022 and December 2023. The control group will proceed with the usual care provided by the (memory) clinics where the patients are recruited. The intervention group will perform a 12-week training intervention according to the “Brain-IT” training concept, in addition to usual care. Global cognitive functioning will be assessed as the primary outcome. As secondary outcomes, domain-specific cognitive functioning, brain structure and function, spatiotemporal parameters of gait, instrumental activities of daily living, psychosocial factors, and resting cardiac vagal modulation will be assessed. Pre- and postintervention measurements will take place within 2 weeks before starting and after completing the intervention. A 2-way analysis of covariance or the Quade nonparametric analysis of covariance will be computed for all primary and secondary outcomes, with the premeasurement value as a covariate for the predicting group factor and the postmeasurement value as the outcome variable. To determine whether the effects are substantive, partial eta-squared (η^2^_p_) effect sizes will be calculated for all primary and secondary outcomes.

**Results:**

Upon the initial submission of this study protocol, 13 patients were contacted by the study team. Four patients were included in the study, 2 were excluded because they were not eligible, and 7 were being informed about the study in detail. Of the 4 included patients, 2 already completed all premeasurements and were in week 2 of the intervention period. Data collection is expected to be completed by December 2023. A manuscript of the results will be submitted for publication in a peer-reviewed open-access journal in 2024.

**Conclusions:**

This study contributes to the evidence base in the highly relevant area of preventing disability because of cognitive impairment, which has been declared a public health priority by the World Health Organization.

**Trial Registration:**

ClinicalTrials.gov NCT05387057; https://clinicaltrials.gov/ct2/show/NCT05387057

**International Registered Report Identifier (IRRID):**

DERR1-10.2196/41173

## Introduction

### Background

Aging is typically accompanied by structural [1,2] and functional [2-4] brain changes associated with a gradual decline in physical [5] and cognitive [1,3,6] abilities. Decline in cognitive functioning exists on a continuum from healthy aging to pathological conditions, such as “mild cognitive impairment” (MCI) or “dementia” [7-9]. MCI has evolved over the last decades [8] and was incorporated in the latest Diagnostic and Statistical Manual of Mental Disorders 5th Edition (DSM-V) and the International Classification of Diseases 11th Revision, as mild neurocognitive disorder (mNCD) [10-13]. Although slightly different definitions have been used in the literature, the core criteria remain [7,8]. According to DSM-V, mNCD is characterized by: “(A) Evidence of modest cognitive decline from a previous level of performance in one or more cognitive domains [...]; (B) cognitive deficits do not interfere with the capacity for independence in everyday activities [...]; (C) Cognitive deficits do not occur exclusively in the context of delirium. (D) Cognitive deficits are not better explained by another mental disorder (eg, major depressive disorder, schizophrenia)” [12]. In individuals with mNCD, deterioration in episodic memory and executive function represent the most prevalent cognitive impairment [14] and are associated with structural [7,15] and functional [7,15,16] brain changes. In addition to cognitive decline, individuals with mNCD may also experience problems in motor function [17,18], impaired balance [18], a higher fall risk [17,19], or difficulties in everyday functioning [20-22]. However, individuals with mNCD retain their capacity for independence in everyday activities [12].

In 2021, over 55 million people were living with major neurocognitive disorder (MNCD; also referred to as “dementia” [12]) [23]. The pooled prevalence of mNCD increases with age and is estimated to be approximately 16% [24], more than twice as high as the prevalence of MNCD [23,25]. The global increase in life expectancy [26] and insufficient levels of physical activity [27] serve as important risk factors for cognitive decline [9,24,28-30] and are expected to boost the incidence and prevalence of mild to major neurocognitive disorders (MNCD). Moreover, individuals with mNCD are at an increased risk of developing dementia. The annual conversion rate of mNCD to MNCD is approximately 4.9 in community settings and 9.6 in specialist clinical settings [31]. Between 14% (clinical population) and 31% (community-based cohort) revert to normal cognitive function [32]. Nonetheless, the pooled progression rate is estimated to be 34%, which is more than twice as high as the pooled reversion rate of 15% [24]. Consequently, the worldwide prevalence of dementia is expected to nearly double over the next 20 years [25]. To counteract this development, the World Health Organization has declared the prevention of disabilities caused by cognitive impairment a public health priority [33].

Individuals with mNCD may represent an optimal target population for pharmacological and nonpharmacological interventions [7]. However, no pharmacological treatment that effectively decelerates or prevents the progression from mNCD to MNCD or decreases the impact of cognitive decline on functioning exists [34-37]. The American Food and Drug Administration recently approved a new but controversial pharmacological agent (aducanumab) to treat individuals with mNCD due to Alzheimer disease (AD) [38,39]. In Europe, Biogen Netherlands BV withdrew its application for marketing authorization of Aduhelm for the treatment of early stages of AD due to insufficient data [40]. Evidence for other pharmacological treatment options (eg, cholinesterase inhibitors, antihypertensive, anti-inflammatory, or lipid-lowering medication, or hormone therapies) and nutritional supplements is largely insufficient and does not support their use for improving cognitive performance, slowing down cognitive decline, or reducing the risk of developing dementia [41-46]. Several nonpharmacological interventions, such as lifestyle changes that target modifiable risk factors such as diabetes mellitus [47-49], hypertension [47-49], obesity [49], depression [49,50], physical [29,49] or cognitive inactivity [30,49], or smoking [49,51], may hold promise for slowing down cognitive decline or reducing the risk of developing dementia [52]. It has been estimated that up to half of the world’s cases of AD, the leading cause of m-MNCDs [12]—may be attributable to these 7 potentially modifiable risk factors [49]. A 10%-25% reduction in these risk factors is estimated to reduce AD prevalence by up to 3 million individual cases worldwide [49]. As physical inactivity is associated with most other modifiable risk factors, an increase in physical activity is believed to have an impact on m-MNCD prevalence [49]. In addition, the theory of cognitive reserve suggests that, “education and mental stimulation throughout life may lower risk of AD and dementia by helping to build a ‘cognitive reserve’ that enables individuals to continue functioning at a ‘normal’ level despite experiencing neurodegenerative changes” [49,53,54]. According to recent network meta-analyses, nonpharmacological interventions targeting modifiable risk factors, such as physical and cognitive exercises, outperform pharmacological therapies [55,56]. It can be hypothesized that a shift from pharmacological to nonpharmacological interventions with multi-domain treatment strategies, including physical exercises and cognitive stimulation, may lead to better results [9,36].

Aerobic training [57-59] and multicomponent physical exercises [57,59,60] are beneficial exercise modes for cognition in patients with NCDs, whereas cognitively engaging exercises appear to have the strongest effect on cognition [61,62]. Combined motor-cognitive training seems to be the most effective type of training for improving cognitive functioning in older adults with mNCD [61,63-65]. These findings are consistent with the “guided-plasticity facilitation” framework [66-68]: physical exercise enhances brain metabolism and promotes neuroplastic processes, whereas these changes in brain plasticity are guided by cognitive stimulation [66,67,69]. Combined motor-cognitive training can be categorized into “sequential,” “simultaneous-additional,” and “simultaneous-incorporated” motor-cognitive training [68]. Incorporating cognitive tasks into motor tasks is more beneficial than “classical” dual-task approaches or sequential motor-cognitive training in terms of stabilizing neuroplasticity effects [68].

Technological innovations (eg, exergames) provide new options to engage older adults with mNCD in (simultaneous-incorporated) motor-cognitive training [70]. “Exergaming is defined as technology-driven physical activities, such as video game play, that requires participants to be physically active or exercise in order to play the game” [71]. When exergames are specifically designed and implemented with a purpose beyond play, it is a “serious game” [72,73]. Exergame-based interventions are highly accepted in individuals with mNCD and increase training adherence and engagement by facilitating training motivation and satisfaction [74]. Furthermore, exergaming offers “the unique opportunity for patients to interact in an enriched environment, providing structured, scalable training opportunities augmented by multi-sensory feedback to enhance skill learning and neuroplasticity through repeated practice” [75], an additional advantage compared with conventional motor-cognitive training. There are consistent positive effects on cognitive functioning, favoring exergaming in people with m-MNCD [74]. Nonetheless, it is currently difficult to draw reliable conclusions about the effectiveness of exergaming in preventing and treating neurocognitive disorders because of the substantial variations in exergame-based training [74]. Gavelin et al [63] synthesized smaller effects on cognitive outcomes for exergaming (standardized mean difference [SMD] 0.13, 95% CI −0.22 to 0.44) compared with sequential (SMD 0.25, 95% CI −0.05 to 0.55) or simultaneous (SMD 0.45, 95% CI 0.11-0.78) motor-cognitive training in individuals with mNCD compared with passive control groups [63]. To the best of our knowledge, 11 studies have investigated exergame-based motor-cognitive training in older adults with mNCD [76-86]. Most of these studies used commercially available exergame systems [76-82], where the training content was not specifically developed for individuals with mNCD. In addition, only one of these studies applied training that individually prescribed content based on a patient’s cognitive abilities [82]. This may explain the small effect findings of Gavelin et al [63], and points to opportunities for improvement in the application of exergames in individuals with mNCD by developing novel exergames and exergame-based training concepts that ensure the implementation of effective training components specifically tailored to the requirements and needs of individuals with mNCD [87]. It seems fair to state that purpose-developed exergames and exergame-based training concepts specifically targeting individuals with mNCD and implementing effective training components will have larger effects on individuals with mNCD.

### Prior Work

We developed an exergame-based motor-cognitive training concept (“Brain-IT”) specifically for older adults with mNCD. It was developed using the “Multidisciplinary Iterative Design of Exergames: A Framework for Supporting the *Design* section, Development, and Evaluation of Exergames for Health” [88]. The target group, therapists, and experts from different fields were involved in the development process to ensure that the training met the requirements and needs of older adults with mNCD and to foster the usability and acceptance of the approach in “real life.” This interactive and participatory design and development process allowed the identification of the key requirements for exergame design as well as the training characteristics. These formed the basis for determining the components of the resulting “Brain-IT” training concept. It ensures the implementation of effective training components and is specifically tailored to the requirements and needs of older adults with mNCD. A detailed description of this design and development process is outlined in a previously published methodological paper that contains our complete training concept with sufficient details to allow full replication [89].

The original “Brain-IT” training concept was already shown to be feasible, usable, and highly accepted in our pilot feasibility randomized controlled trial (RCT), including a small sample of older adults with mNCD (n=18; under peer review; registered at ClinicalTrials.gov NCT04996654). Minor modifications were incorporated to further optimize the “Brain-IT” training concept, making it applicable for the systematic evaluation of effectiveness in samples of older adults with mNCD.

### Explanation and Choice of Comparators

According to a recent systematic review summarizing worldwide available clinical practice guidelines and consensus statements, recommendations for the treatment and management of individuals with mNCD can be classified into 4 categories: interventions for risk reduction, pharmacological interventions, nonpharmacologic interventions, and counseling. Recommendations for nonpharmacological interventions mainly include physical and cognitive activity interventions; however, the specific training characteristics (eg, frequency, intensity, type, volume, and progression) are not specified [90].

In line with these recommendations, in Switzerland, usual care for mNCD typically includes treating medical conditions other than mNCD (eg, diabetes mellitus and depressive symptoms), controlling comorbidities (eg, hypertension and obesity), and managing risk factors (eg, smoking habits and physical and cognitive inactivity). In this regard, usual care may include medication, recommendations for changing lifestyle habits (eg, living a cognitively, physically, and socially active life), physiotherapy to treat specific health problems, such as back pain or mobility problems, occupational therapy, or day clinic visits. This justifies the selection of usual care as the control intervention.

### Objectives and Hypotheses

This study explores the effectiveness of the “Brain-IT” training in improving global cognitive functioning in older adults with mNCD. With this, we aim to obtain a sufficiently precise estimate of the treatment effect to minimize the sample size needed for a future full-scale RCT.

- Null Hypothesis (H_0_0): In older adults with mNCD, the addition of the “Brain-IT” training to usual care has no significant effect on global cognitive functioning compared with usual care.
- Alternative Hypothesis (H_A_): In older adults with mNCD, the addition of the “Brain-IT” training to usual care results in differing effects on global cognitive functioning compared with usual care.

As secondary objectives, the effects of the “Brain-IT” training on (a) domain-specific cognitive functioning (ie, learning and memory, complex attention, executive function, and visuospatial skills), (b) brain structure and function, (c) spatiotemporal parameters of gait, (d) instrumental activities of daily living (IADL), and (e) psychosocial factors (ie, QoL [quality of life], and levels of depression, anxiety, and stress), and (f) cardiac vagal modulation (ie, resting vagally-mediated heart rate variability [vm-HRV]) in older adults with mNCD as compared with usual care will be explored. (B) Brain structure and function will be evaluated to explore the possible underlying neural changes in training in relation to adaptations in cognitive performance. The following hypotheses are formulated for the remaining outcomes:

- Null Hypothesis (H_0_): In older adults with mNCD, the addition of the ‘Brain-IT’ training to usual care has no significant effect on (a) domain-specific cognitive functioning, (c) spatiotemporal parameters of gait, (d) IADL and (e) psychosocial factors (ie, QoL, and levels of depression, anxiety, stress), and (f) cardiac vagal modulation (resting vm-HRV) compared with usual care.
- Alternative Hypothesis (H_A_): In older adults with mNCD, the addition the “Brain-IT” training to usual care results in differing effects on (a) domain-specific cognitive functioning, (c) spatiotemporal parameters of gait, (d) IADL, (e) psychosocial factors (ie, QoL, and levels of depression, anxiety, and stress), and (f) cardiac vagal modulation (resting vm-HRV) compared with usual care.

The rationale and description of the specific hypotheses for (c) spatiotemporal parameters of gait are explained in more detail in the section “Methods—Outcomes—Secondary Outcomes—Spatiotemporal Parameters of Gait.”

## Methods

This study protocol (version 1.0; July 20, 2022) was developed in accordance with established guidelines from the “SPIRIT 2013 Statement: Defining Standard Protocol Items for Clinical Trials” [91,92] (see the checklist in Multimedia Appendix 1).

### Ethics Approval

All the study procedures will be performed in accordance with the Declaration of Helsinki. The study protocol was approved by the Ethics Committees of Zurich and Eastern Switzerland (EK-2022-00386).

### Trial Design and Study Setting

A 2-arm, prospective, parallel-group, single-blinded (ie, outcome evaluator of pre- and postintervention measurements blinded to group allocation) RCT with a 1:1 allocation ratio (ie, intervention: control), including 34 to 40 older adults with mNCD, will be conducted between May 2022 and December 2023. The control group will proceed with usual care as provided by the (memory) clinics where the patients are recruited, while the intervention group will perform a 12-week training intervention according to the “Brain-IT” training concept in addition to usual care (see section “Methods—Interventions”). The study was registered at clinicaltrials.gov before the start of patient recruitment (NCT05387057; date of registration: May 18, 2022; see Table 1 for details) and will be reported according to “The Consolidated Standards of Reporting Trials (CONSORT) 2010 statement” [93] and elaboration paper [94]. The study setup is multicentric (Zurich and St. Gallen) and national (Switzerland).

**Figure 1:**
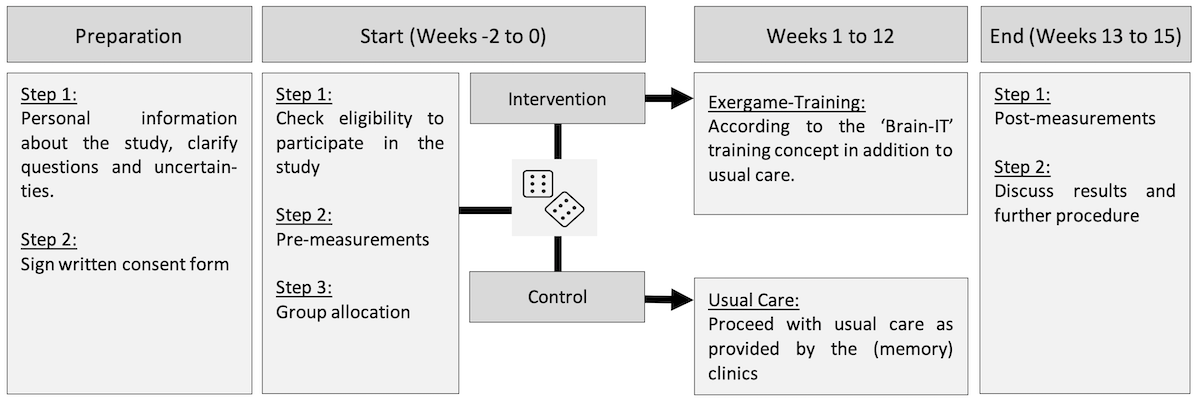
Graphical overview of the study procedures. The cubes are used to illustrate the randomization process (variable block randomization (ie, block sizes=4, 6, 8) with a 1:1 allocation ratio stratified by sex and per institute (study center), as described in section “Methods–Randomization”).

After recruitment and providing written informed consent (see section “Methods—Recruitment”), participants will be screened on eligibility (see section “Methods—Eligibility Criteria”), and premeasurements will be scheduled for all eligible participants. Pre- and postintervention measurements will take place within 2 weeks before starting and after completing the intervention. For participants recruited in Zurich, preintervention measurements will take place at ETH Hönggerberg (Robert-Gnehm-Platz 1, CH-8093 Zurich). For participants recruited in St. Gallen, premeasurements will take place at the Eastern Switzerland University of Applied Sciences (Vadianstrasse 29, CH-9000 St Gallen). The measurements will take approximately 90 minutes. For all participants with no contraindications to magnetic resonance imaging (MRI), an additional appointment to conduct an MRI scan (duration: approximately 1 hour [including preparation]) at University Hospital Zurich (Rämistrasse 100, CH-8006 Zurich) will be scheduled. All measurements will be led by 2 investigators from our research team trained in the application of the measurement techniques and protocols. Pre- and postintervention measurements will be scheduled to occur at approximately the same time of the day (±2 h) for each participant. To minimize the influence of transient confounding effects on HRV, all participants will additionally be instructed verbally and in writing to follow a normal sleep routine the day before the experiment, to avoid intense physical activity and alcohol consumption within 24 hours before measurements, and to refrain from coffee–or caffeinated drinks as well as food consumption at least 2 hours before measurements [95]. After completing premeasurements, participants will be randomly allocated to the intervention or control group and will be instructed about their respective intervention procedures (see section “Methods—Interventions”). For participants in the intervention group, the exergame device will be installed in their homes; they will receive safety instructions and will be familiarized with the exergame training system. Subsequently, the study intervention will be started (see section “Methods—Interventions—Intervention Group”). After completing the 12-week intervention period, postintervention measurements will be performed for both groups. No compensation will be granted to the participants, but detailed feedback on individual performance as well as the study outcomes in general will be provided at the end of the trial. Figure 1 summarizes the study procedures.

**Table 1:** Overview of trial registration data.

Data category	Information
Primary registry and trial identifying number	ClinicalTrials; NCT05387057
Date of registration in primary registry	May 18, 2022
Secondary identifying numbers	N/A^a^
Sources of monetary or material support	Synapsis Foundation—Dementia Research Switzerland
Sponsor-investigator	Prof Dr Eling D. de Bruin, Motor Control and Learning Group – Institute of Human Movement Sciences and Sport, Department of Health Sciences and Technology, ETH Zurich, Leopold-Ruzicka-Weg 4, Zurich, Switzerland, eling.debruin@hest.ethz.ch and Department of Health, OST – Eastern Swiss University of Applied Sciences, Vadianstrasse 29, St. Gallen, Switzerland, eling.debruin@ost.ch
Contact for public queries	Patrick Manser; Motor Control and Learning Group – Institute of Human Movement Sciences and Sport, Department of Health Sciences and Technology, ETH Zurich, Zurich, Switzerland; patrick.manser@hest.ethz.ch
Contact for scientific queries	Patrick Manser; Motor Control and Learning Group – Institute of Human Movement Sciences and Sport, Department of Health Sciences and Technology, ETH Zurich, Zurich, Switzerland; patrick.manser@hest.ethz.ch
Public title	Effectiveness of a Novel Exergame-Based Training Concept for Older Adults with Mild Neurocognitive Disorder
Scientific title	Effectiveness of an Individualized Exergame-based Motor-Cognitive Training Concept Targeted to Improve Cognitive Functioning in Older Adults with Mild Neurocognitive Disorder–A Randomized Controlled Trial
Countries of recruitment	Switzerland
Health conditions or problems studied	Older adults with mild neurocognitive disorder
Interventions	Control group: The control group will proceed with usual care as provided by the (memory) clinics where the patients are recruited. Intervention group: Participants in the intervention group will perform a 12-week training intervention according to the newly developed “Brain-IT” training concept in addition to their usual care.
Key inclusion and exclusion criteria	Key inclusion criteria: (1=mNCD) clinical diagnosis of “Mild Neurocognitive Disorder” OR (2=sMCI) patients sMCI German speaking Able to stand at least for 10 min without assistance Exclusion criteria: Mobility impairments that prevent experiment participation Presence of additional, clinically relevant (ie, acute or symptomatic) neurological disorders Presence of any other unstable or uncontrolled diseases (eg, uncontrolled high blood pressure, progressing or terminal cancer)
Study type	Study type: Interventional Primary purpose: Prevention Study phase: N/A Interventional study model: Randomized, Parallel Assignment Masking: Single blind
Date of first enrollment	June 22, 2022
Target sample size	40
Recruitment status	Recruiting
Primary outcomes	Changes in global cognitive functioning (Time Frame: both, the pre- and the postintervention measurements of the primary outcome will take place within 2 weeks before starting or after completing the intervention)
Key secondary outcomes	Changes in Learning and Memory Changes in Complex Attention Changes in Executive Function Changes in Visuospatial Skills Changes in Brain Structure and Function Changes in Spatiotemporal Gait Parameters Changes in instrumental activities of daily living Changes in Psychosocial Factors Changes in vagally-mediated Heart Rate Variability For all secondary outcomes: (Time Frame: both, the pre- and postintervention measurements of the primary outcome measurement will take place within 2 weeks before starting or after completing the intervention)

^a^N/A: not applicable.

### Recruitment

Older adults with mNCD will be recruited between May 2022 and September 2023 in collaboration with (memory) clinics in a larger area of Zurich and St. Gallen. Suitable patients will either be identified from medical records and patient registries of (memory) clinics, or from recent diagnostics performed by medical doctors or therapists authorized to search for medical records. Alternatively, suitable patients will be identified by an informant (ie, health care professionals) based on suspicion of MCI in one of their patients (see *Methods—Eligibility Criteria* section). Identified patients will be verbally informed about the study and will receive leaflets by their physicians or therapists containing key information about study participation and researchers’ contact details. If patients are interested in being informed about the study in detail, they will be asked to provide consent to share their contact details with the research team and will be contacted by phone or email by a trained investigator of the study team. In case of initial interest in participating in the study, all interested subjects will be fully informed about the study procedures in-person (at the interested persons’ home or at one of the study centers, depending on their preferences) by providing verbal explanations and an information sheet. After sufficient time for consideration (ie, at least 24 hours after handing out the study information sheet, but on average around 1 week), suitable patients willing to participate in the study will provide written informed consent in a second in-person meeting with one of the trained investigators of the study team at the home of the interested persons or at one of the study centers. After providing written informed consent, participants will be fully screened for eligibility (see section “Methods—Eligibility Criteria”), and premeasurements will be scheduled.

### Eligibility Criteria

All eligibility criteria are detailed in Textbox 1.

**Description of all eligibility criteria.**

Inclusion criteria

- Participants fulfilling all the following inclusion criteria were eligible:
  (1=mild neurocognitive disorder) clinical diagnosis of “mild neurocognitive disorder” according to International Classification of Diseases 11th Revision [13] or Diagnostic and Statistical Manual of Mental Disorders 5th Edition [12] OR (2=screened for mild cognitive impairment [sMCI]) patients sMCI according to the following criteria: (1) informant (ie, health care professional)-based suspicion of MCI confirmed by (2) an objective screening of MCI based on the German version of the using the quick mild cognitive impairment screen [96] with (2A) a recommended cut-off score for cognitive impairment (MCI or dementia) of <62/100 [97], while (2B) not falling below the cut-off score for dementia (ie<45/100 [97]).
  Speaking German
  Able to stand at least for 10 minutes without assistance

Exclusion criteria

- The presence of any of the following criteria led to exclusion:
  Mobility impairments (ie, gait and balance) that prevent experiment participation
  Presence of additional, clinically relevant (ie, acute or symptomatic or both) neurological disorders (ie, epilepsy, stroke, multiple sclerosis, Parkinson’s disease, brain tumors, or traumatic disorders of the nervous system)
  Presence of any other unstable or uncontrolled diseases (eg, uncontrolled high blood pressure and progressing or terminal cancer)
- COVID-19–specific risk factors (according to the Swiss Federal Office of Public Health) are additional exclusion criteria. In case of COVID-19–specific exclusion criteria, participation in the study will only be allowed when the patients’ treating physician provides written informed consent allowing participation in the study despite the presence of COVID-19–specific exclusion criteria. COVID-19–specific exclusion criteria include:
  High blood pressure (self-reported; systolic ≥140 mm Hg or diastolic ≥90 mm Hg)
  Chronic respiratory condition
  Uncontrolled type 2 diabetes
  Condition or therapy that weakens the immune system
  Unstable cardiovascular disease
  Cancer (present or under treatment)
  Serious obesity (BMI≥40 kg/m^2^)

### Interventions

#### Control Group

The control group will proceed with the usual care provided by the (memory) clinics where the patients are recruited. As described in section “Introduction—Choice of comparators” and based on clinical practice guidelines [90], in Switzerland, usual care is highly individual, varies between (memory) clinics where patients are recruited, and it is unclear whether patients comply with the recommendations of their clinicians. Therefore, details about all structured or guided usual care activities or both, as well as medication intake, will be assessed in both the intervention and control groups. If there are relevant between-group differences in usual care, these differences will be accounted for in the analysis and discussion of results.

#### Intervention Group

Participants in the intervention group will perform a 12-week training intervention in addition to their usual care (as provided by the [memory] clinics where patients were recruited). The training will be prescribed according our “Brain-IT” training concept. This motor-cognitive training concept represents a guideline for applying exergame-based motor-cognitive training by standardizing the training characteristics (eg, training frequency, intensity, and duration) as well as the structure and content of the training, whereas the exergame device and the specific games used within each of the defined neurocognitive domains can be replaced by alternative exergames. In this project, our training concept was implemented using the “Senso (Flex)” (Dividat AG). This platform was found to be suitable for the “Brain-IT” training [87] and is a widely used means for motor-cognitive training within geriatric populations, physiotherapy, and rehabilitation in Switzerland. The original “Brain-IT” training concept has recently been published with sufficient detail about the exergame components as well as the exercise and training characteristics (ie, including all predefined levels of task demands as well as the detailed progression rules) to allow full replication (ie, consider supplementary file 3 of [89]). Some minor modifications were implemented in the “Brain-IT” training concept for use in this study, based on the findings of our pilot feasibility RCT (under peer review; registered at ClinicalTrials.gov NCT04996654). This will be complemented in cases in which suitable new games become available throughout the study. The final (modified) “Brain-IT” training concept will be published with the manuscript reporting the results of this study, and will additionally provide specific information that would theoretically allow adaptation of the training to other hardware and software solutions. To ensure replicability, the “Brain-IT” training concept was planned and will be reported using the Consensus on Exercise Reporting Template [98].

**Figure 2:**
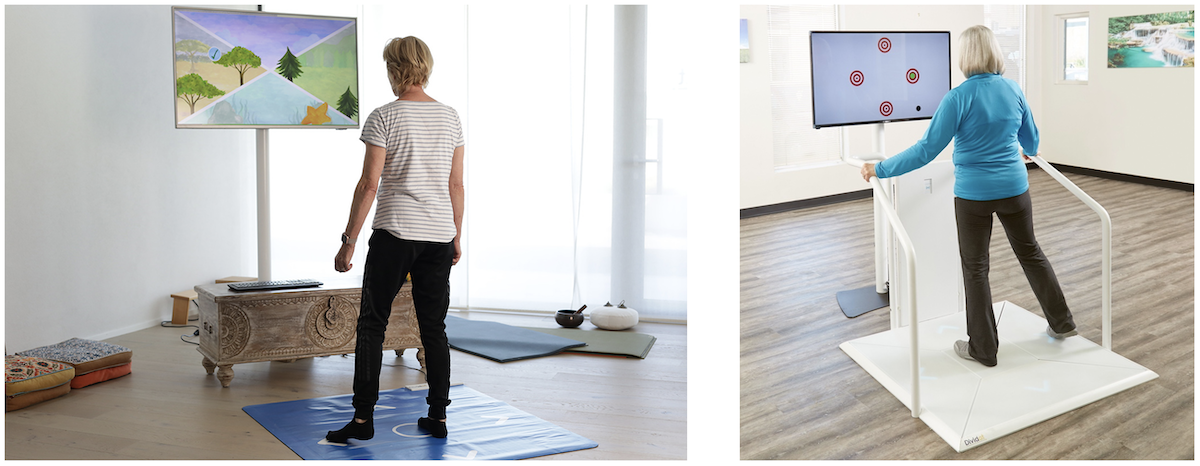
Exergame Device used as means to implement the “Brain-IT” training concept in this study: “Senso Flex” for home-based use (left side) and its stationary version (“Senso” for stationary use in physiotherapies, nursing homes, or rehabilitation clinics (right side). Photos provided by Dividat AG.

For an overview, the “Brain-IT” training concept consists of individually adapted multidomain exergame–based simultaneous motor-cognitive training with incorporated cognitive tasks that is adopted with a deficit-oriented focus on the neurocognitive domains of (1) learning and memory, (2) executive function, (3) complex attention, and (4) visuospatial skills. Each participant is instructed to train 5 times per week for 24 minutes per session, resulting in a weekly training volume of 120 minutes. All training sessions are planned to take place at participant’s homes using the “Senso Flex” hardware (Dividat AG; CE certification pending; see left side of Figure 2). The “Senso Flex” is a home-based version of the “Senso” (Dividat AG; CE certification; see right side of Figure 2). It consists of a 1.11×0.99 m rollable mat that is plugged into a portable computer and a frontal television (or other screen) at home. Both systems divide the pressure-sensitive stepping area into five fields: (1) center (home position), (2) front, (3) right, (4) back, and (5) left. The device detects the participants’ position and timing of movements to interact with different game scenarios programmed in the training software. Weight shifting, walking on the spot, and steps in 4 directions (ie, front, right, back, and left) enable interaction with and control of the exergame scenarios displayed on a frontal screen. Visual, auditory, and somatosensory (vibrating platform; only available on the “Senso”) feedback is provided in real time to enrich the game experience. Various games are available for training different neurocognitive domains (more details on how the device is implemented in our training concept have been provided [89]).

Adherence to the training will be monitored (see section “Outcomes—Other Outcomes—For the intervention group,” ie, exergame training), and participants will be actively motivated and reminded to adhere to the training by the person responsible for supervision and correspondence with the respective study participant. Upon the request of participants, training can be paused for a maximum of 2 weeks (eg, because of holidays).

### Outcomes

#### Overview

To ensure comparability of the study outcomes, global cognitive functioning will be assessed as the primary outcome. As secondary outcomes, domain-specific assessments evaluating the key neurocognitive domains (as defined in [10] in line with DSM-V [12] and according to recommendations [7]) of (1) learning and memory; (2) complex attention; (3) executive function; and (4) visuospatial skills, as well as brain structure and function, spatiotemporal parameters of gait, IADL, psychosocial factors (ie, QoL, and levels of depression, anxiety, and stress), and cardiac vagal modulation (resting vm-HRV) will be assessed. Table 2 provides an overview of all endpoints.

**Table 2:** Overview of all primary and secondary outcome measures, outcome variables and interpretation guide.

Outcome measures			Outcome variables		Interpretation guide
**Primary**					
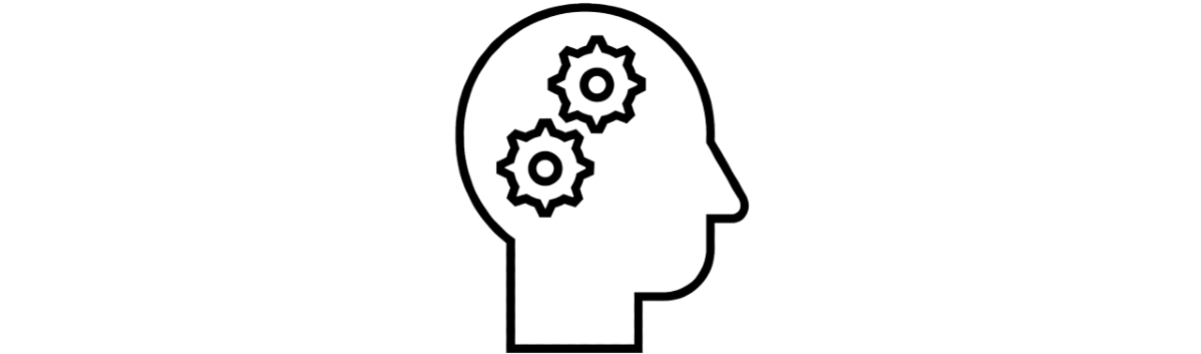			Global cognitive functioning		
	Quick mild cognitive impairment screen [96,99]	Total point score		Improvement=↑^a^	
**Secondary**					
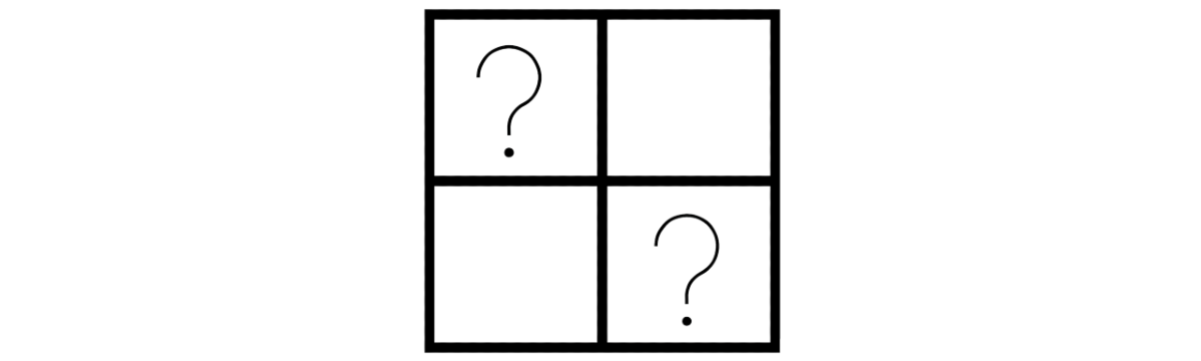			Learning and memory		
	Subtest “logical memory” of the Wechsler Memory Scale—fourth edition [100,101]	Total point score part 1—free recall		Improvement=↑	
	Subtest “logical memory” of the Wechsler Memory Scale—fourth edition [100,101]	Total point score part 2—free recall		Improvement=↑	
	Subtest “logical memory” of the Wechsler Memory Scale—fourth edition [100,101]	Total point score part 2—recognition		Improvement=↑	
	PEBL^b^ digit span forward [102-104]	Total point score		Improvement=↑	
	PEBL digit span forward [102-104]	Maximum span		Improvement=↑	
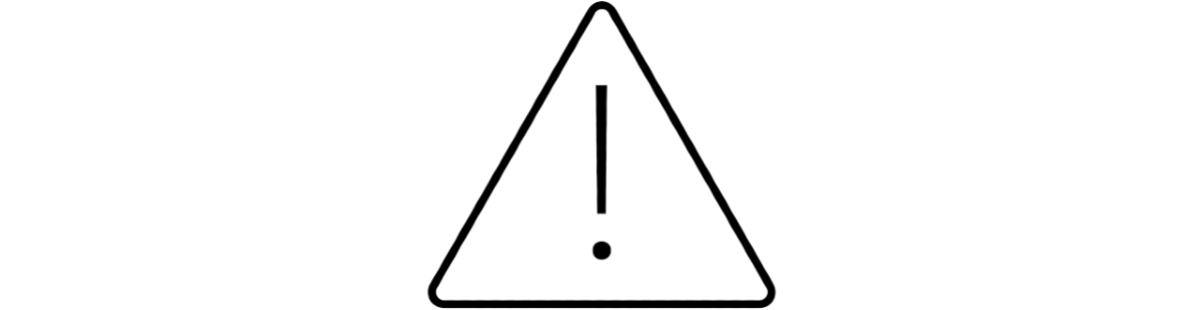			Complex attention		
	PEBL trail making test—part A [102]	Completion times		Improvement=↓^c^	
	PEBL trail making test—part A [102]	Number of errors		Improvement=↓	
	Subtest “Go-NoGo” of the test of attentional performance [105]	Median reaction time (ms)		Improvement=↓	
	Subtest “Go-NoGo” of the test of attentional performance [105]	Number of errors		Improvement=↓	
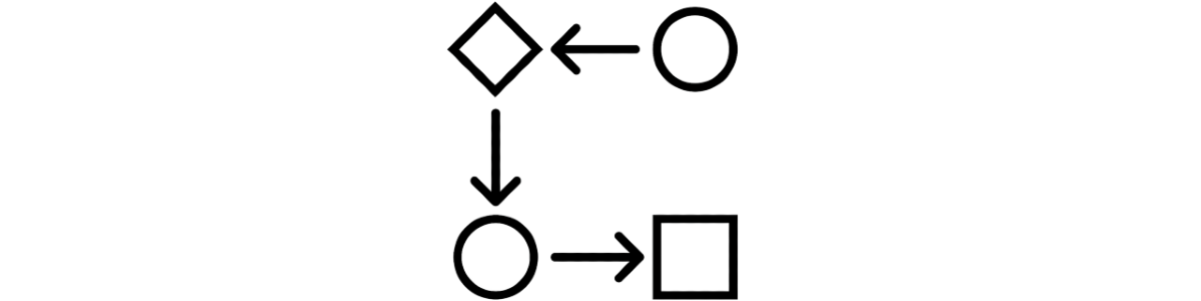			Executive function		
	HOTAP picture-sorting test part A [106]	Combi score (ie, sum of the points divided by the time they needed to arrange the cards) (points×min^−1^)		Improvement=↑	
	PEBL digit span backward [102-104]	Total point score		Improvement=↑	
	PEBL digit span backward [102-104]	Maximum span		Improvement=↑	
	PEBL trail making test—part B [102,104]	Completion time (s)		Improvement=↓	
	PEBL trail making test—part B [102,104]	Number of errors		Improvement=↓	
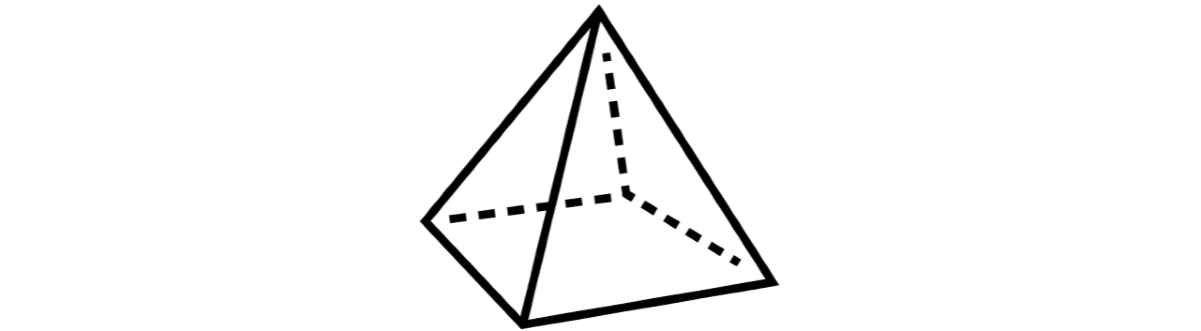			Visuospatial skills		
	PEBL-Mental Rotation Task [102,104,107]	Median reaction time of correct answered trials (ms)		Improvement=↓	
	PEBL-Mental Rotation Task [102,104,107]	Performance (number of correct answered trials)		Improvement=↑	
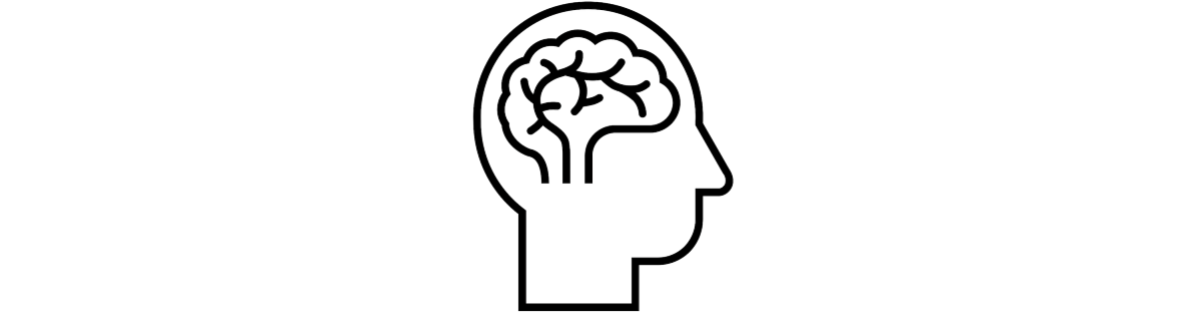			Brain Structure and Function		
	3D isotropic T1-weighted MRI^d^ scan	Gray matter volume of defined key regions of interest (mm^3^)		Improvement=↑	
	3D isotropic T1-weighted MRI scan	White matter volume of defined key regions of interest (mm^3^)		Improvement=↑	
	Diffusion Tensor Imaging MRI scan	Mean diffusivity of defined key regions of interest		Improvement=↓	
	Diffusion Tensor Imaging MRI scan	Fractional anisotropy of defined key regions of interest		Improvement=↑	
	Resting state T*2-weighted blood oxygen level-dependent fMRI^e^	Individual functional connectivity maps between the hippocampal seed and the defined key regions of interest		Improvement=↑	
	Task-based T*2-weighted blood oxygen level-dependent fMRI	Individual functional connectivity maps between the hippocampal seed and the defined key regions of interest		Improvement=↑	
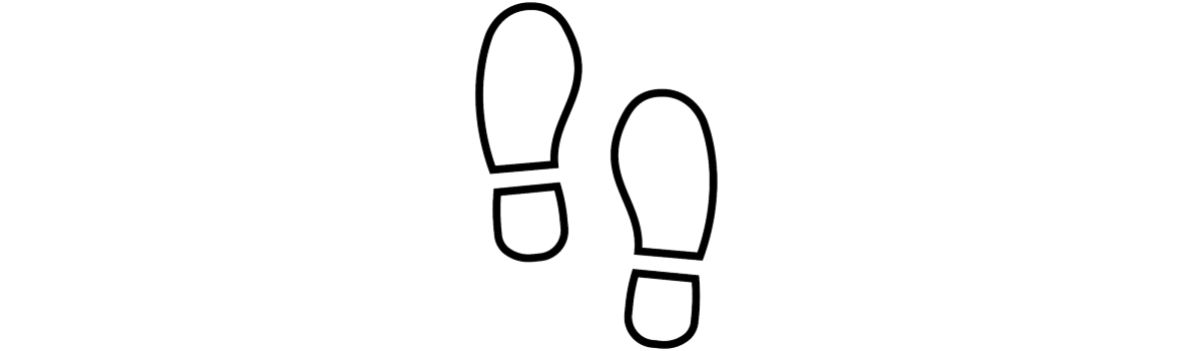			Spatiotemporal parameters of gait		
	Gait analysis^f^ [108]	Walking speed (m/s)		Improvement=↑	
	Gait analysis	Stride duration (ms)		Improvement=↓	
	Gait analysis	Stride length (cm)		Improvement=↑	
	Gait analysis	Stance phase duration (ms)		Improvement=↓	
	Gait analysis	Swing time (ms)		Improvement=↓	
	Gait analysis	Single support time (ms)		Improvement=↓	
	Gait analysis	Double support time (ms)		Improvement=↓	
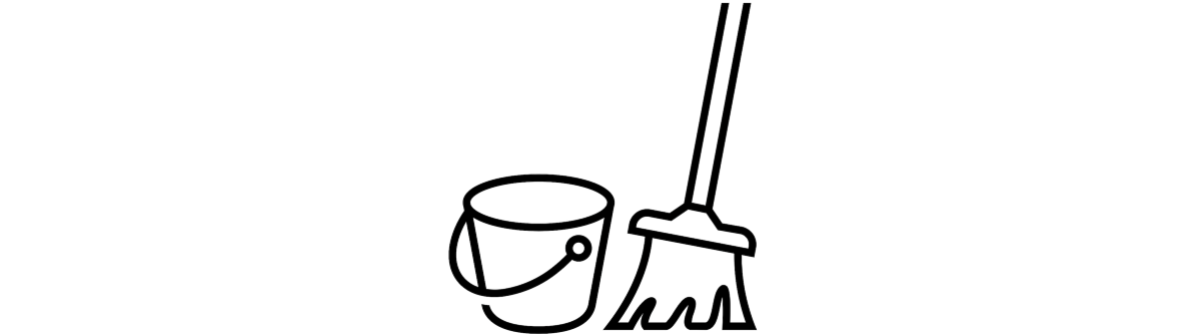			IADL^g^		
	Amsterdam IADL questionnaire [109]	T- score		Improvement=↑	
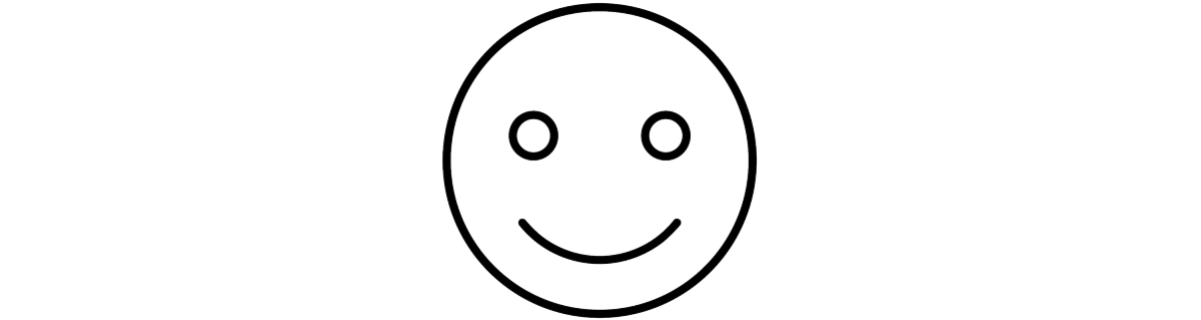			Psychosocial factors		
	Quality of life-Alzheimer disease [110-112]	Overall score		Improvement=↑	
	Depression, anxiety and stress scale-21 [113-117]	Overall score—subscale depression		Improvement=↓	
	Depression, anxiety and stress scale-21 [113-117]	Overall score—subscale anxiety		Improvement=↓	
	Depression, anxiety and stress scale-21 [113-117]	Overall score—subscale stress		Improvement=↓	
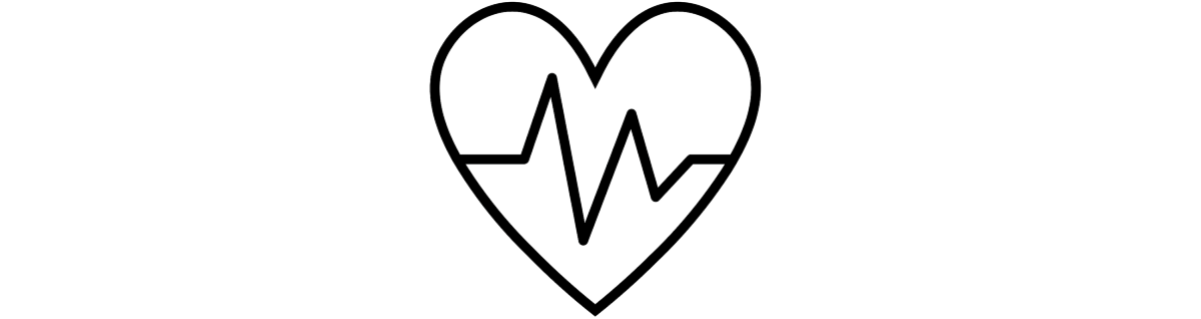			Resting vagally–mediated Heart Rate Variability		
	resting vm-HRV^h^ measurement^i^ [118]	Mean R-R time interval (ms)		Improvement=↑	
	Resting vm-HRV measurement	Root Mean Square of successive RR interval differences		Improvement=↑	
	Resting vm-HRV measurement	Percentage of successive RR intervals that differ by more than 50 ms (%)		Improvement=↑	
	Resting vm-HRV measurement	Absolute power of the high-frequency (0.15-0.4 Hz) band (ms^2^)		Improvement=↑	
	Resting vm-HRV measurement	Relative power of the high-frequency (0.15-0.4 Hz) band (nu)		Improvement=↑	
	Resting vm-HRV measurement	Poincaré plot SD perpendicular to the line of identity (ms)		Improvement=↑	
	Resting vm-HRV measurement	Parasympathetic nervous system tone index		Improvement=↑	

^a^Higher values or an increase over time indicate better functioning or improvement in the respective study endpoint.

^b^PEBL: psychology experiment building language.

^c^Lower values or a decrease over time indicate better functioning or improvement in the respective study endpoint.

^d^MRI: magnetic resonance imaging.

^e^fMRI: functional magnetic resonance imaging.

^f^Instrumented gait analysis using a figure of 8 walking path [108] at preferred walking speed using BTS G-WALK (BTS Bioengineering SpA, Garbagnate Milanese, Italy) inertial sensor attached with semielastic belt to the lower back of the participant.

^g^IADL: instrumental activities of daily living.

^h^vm-HRV: vagally-mediated heart rate variability.

^i^5 min resting vm-HRV measurement with heart rate monitor (Polar M430) and sensor (Polar H10) analyzed using Kubios HRV Premium (Kubios Oy, Kuopio, Finland, version 3.4) [118].

#### Primary Outcome

The German version of the quick mild cognitive impairment (Qmci) screen will be used to assess global cognitive functioning [96,99]. The Qmci has been validated against the standardized Alzheimer’s Disease Assessment Scale-Cognitive Subscale (ADAS-cog) [96,119], which is considered the gold standard for assessing the efficacy of antidementia treatments [120-122]. The Qmci was shown to “correlate strongly, significantly and correspondingly over time to the standardized ADAS-cog and that both are equally sensitive with similar responsiveness to deterioration over time” [119], “suggesting that the Qmci could be substituted for a more detailed neuropsychological instrument in clinical trials” [96,119]. Furthermore, it is accurate in differentiating mNCD from normal cognition and MNCD [119]. In this regard, it has also been externally validated and has shown a higher level of accuracy, sensitivity, and specificity than commonly used tests such as the (Standardised) Mini Mental State Exam ([S]MMSE) and the Montreal Cognitive Assessment for detecting cognitive impairment (MCI and dementia) [123]. In comparison to the (S)MMSE and Montreal Cognitive Assessment, the Qmci includes a more detailed scoring system and a logical memory task that allows the Qmci to detect more subtle cognitive abnormalities and avoid ceiling effects [123]. Qmci was scored as the point rate out of a maximum score of 100. It consists of 6 subtests: orientation (10 points), registration (5 points), clock drawing (15 points), delayed recall (20 points), verbal fluency (20 points), and logical memory (30 points) [99,124]. Qmci will be administered and evaluated according to published guidelines [99].

To the best of our knowledge, the smallest unit of clinically meaningful change has not yet been determined for the Qmci. However, changes in Qmci scores were very similar to changes in standardized ADAS-cog scores. Longitudinal data of more than 360 patients with m-MNCD were analyzed for responsiveness of the Qmci and the standardized ADAS-cog [125] by calculating standardized response mean from baseline to scores obtained at 1, 3, 6, 9, and 12 months. The mean change between months 1 and 12 was 5 points (SD 7.56) for the standardized ADAS-cog scores, and 5.41 points (SD 10.02) for the Qmci. The paired-samples *t* test showed no statistically significant difference in standardized response means for standardized ADAS-cog and Qmci (t_357_=0.32; *P*=.75) [119]. Therefore, it is anticipated that the smallest units of clinically meaningful changes between these 2 measures are similar. For the standardized ADAS-cog, the minimal clinically relevant change is estimated to be 3 points for individuals with mNCD compared with ≥4 points based on previous recommendations made by a consensus committee of the Food and Drug Administration for patients in more advanced stages [126,127]. Therefore, it is assumed that a change of ≥3 points in the Qmci score can be regarded as a clinically meaningful change.

#### Secondary Outcomes

##### Learning and Memory

Learning and memory will be assessed using the German version of the subtests “logical memory” of the Wechsler Memory Scale—fourth edition (WMS-IV-LM) [100,101] and a computerized version of the digit span forward (DSF) test (psychology experiment building language [PEBL; PEBL-DSF]) [102-104].

The WMS-IV-LM measures auditory verbal contextual learning and memory with excellent reliability and validity [101]. The validated older adults battery (aged 65 years or older) of the German version of the WMS-IV-LM [100,101,128] will be used for all participants. The test will be instructed, conducted, and evaluated according to the standardized administration and scoring manual [100]. During the 20 to 30 minutes retention phase, unrelated assessments (eg, gait analysis, questionnaires) will be performed that do not interfere with memory.

The PEBL-DSF test assesses immediate recall and will be executed using the PEBL Test battery software [version 2.1 (2), with default settings] [102-104]. Participants were required to remember and repeat the digit sequences presented on the screen. Span length covers 2 to a maximum of 8 digits. For each digit span, 2 trials will be presented before increasing the sequence length (in case at least one of the 2 trials is completed correctly). For every correct replication of a digit sequence, 1 point will be scored, summing up to a total point score. In addition, the length of the longest correctly repeated digit sequence will be recorded as the maximum span. Instructions will be presented on the screen and will be explained verbally to each participant before starting the test.

##### Complex Attention

Complex attention will be assessed using a computerized version of the Trail Making Test—Part A (PEBL-TMT-A) [102,104] and the subtest “Go-NoGo” of the Test of Attentional Performance (test of attentional performance [TAP] Go-NoGo) [105].

The TMT-A is a valid and reliable neuropsychological test for assessing psychomotor processing speed and visuo-perceptual abilities [129-134]. A computerized version of the TMT-A PEBL Test battery software [version 2.1 (2), with default settings] will be used [102,104,135]. Participants will be verbally instructed, and a short practice session will be conducted before starting the test. The completion time will be limited to 300 seconds. Completion times (seconds; including time for error correction) and the number of errors will be measured.

The TAP (version 2.3.1, PSYTEST, Psychologische Testsysteme, Herzogenrath, Germany) is a valid and reliable computerized test battery to assess various attentional and executive functions [105,136], with norm values for healthy older adults provided by the supplier [105,137]. The TAP Go-NoGo will be used to assess selective attention and inhibition. The test form “1 of 2” will be instructed, conducted and evaluated according to the standardized protocol of the manufacturer. The median reaction time and number of errors will be measured [105].

##### Executive Function

Executive function will be assessed considering planning (ie, using the HOTAP picture-sorting test part A [106]), working memory (ie, using a computerized version of the Digit Span Backward test; PEBL Digit Span Backward [PEBL-DSB]) [102-104], and cognitive flexibility (ie, using a computerized version of the Trail Making Test-Part B [PEBL-TMT-B] [102,104]).

HOTAP picture-sorting test part A [106] will be used to measure planning ability. A set of photo cards containing actions typical of everyday life (eg, making coffee, washing clothes, and grocery shopping) will be presented. Participants will be verbally instructed to sort photo cards on which the individual substeps of these typical everyday actions are depicted. The test will be conducted according to the manufacturer’s standardized protocol. The outcome variable is a combination score calculated as the sum of points divided by the time needed to arrange the cards [106].

The PEBL Digit Span Backward (PEBL-DSB) [102-104] test will be used to assess the short-term working memory capacity. It will be instructed, administered, and scored identical to the PEBL-DSF, but participants will have to remember and repeat the digit sequences in reverse order.

The TMT-B is a valid and reliable neuropsychological test for assessing cognitive flexibility [129-134]. It consists of 25 randomly allocated circles distributed over a sheet of paper. A computerized version of the TMT-A (PEBL Test battery software [version 2.1 (2), with default settings]) will be used [102,104,135]. It will be instructed, administered, and scored identical to the PEBL-TMT-A.

##### Visuospatial Skills

Visuospatial skills will be tested using a computerized version of the classic Shepard and Metzler mental rotation task [138]. The PEBL-Mental Rotation Task (PEBL-MRT) will be executed using PEBL Test battery software [version 2.1 (2), with default settings] [102,104,107]. Instructions will be presented on the screen and will be explained verbally to each participant before starting the task. Pairs of differently rotated 2D polygons will be presented simultaneously on the screen. Participants need to decide as quickly as possible whether the 2 presented objects are identical (ie, pressing <Lshift> on the keyboard) or different (ie, pressing <Rshift> on the keyboard). Median reaction time of correct answered trials as well as performance (number of correct answered trials) are an indicator for mental rotation ability [107,138]. Trials with reaction times less than 0.2 seconds or greater than 13 seconds will be excluded from the data analysis [107].

##### Brain Structure and Function: Data Acquisition

Brain structure and function will be evaluated by MRI using a 3.0 Tesla Philips whole-body scanner (Philips Medical Systems, Best, and Netherlands) to explore possible underlying neural changes of training in relation to adaptations in cognitive performance. Only participants having no contraindications to MRI (ie, any MRI-incompatible metallic parts within the body, metallic or electronic implants [eg, heart pacemaker, brain pacemaker, and cochlear implants], and strong claustrophobia) will be measured. During measurements (duration=approximately 35 min), participants will lay comfortably in the MRI scanner and will be asked to avoid head movements. Data will be collected according to the Canadian Dementia Imaging Protocol (CDIP) [139] for comparison with other studies. The CDIP was developed to harmonize MRI acquisitions in the context of studying primary and secondary causes of morbidity of neurodegeneration in a wide range of neurological pathologies related to aging [139]. First, the following parts of the core CDIP will be applied: anatomical imaging (1.) and connectivity and functional imaging (2. – 3.) [139]:

- a 3D isotropic t1-weighted (*t*_1_w) scan (duration=6.5 minutes) for assessing fine anatomical detail and brain atrophy (voxel size=1.0×1.0×1.0 mm^3^) with an acceleration factor of 2 (TFE-Sense: 2);
- diffusion tensor imaging (DTI; duration=6 minutes) for assessment of white matter microstructural integrity and connectivity, with resolution 2.0×2.0×2.0 mm^3^, a minimum of 30 uniformly distributed directions with b=1000 second/mm^2^, (EPI-Sense 2-32 directions; we use the vendor-provided directions set), and an acceleration factor of 2; and
- a resting state fMRI (duration=9 minutes) for assessment of functional networks and pathways using a T*_2_-weighted blood oxygen level-dependent-sensitive sequence, with resolution of 3.5×3.5×3.5 mm^3^, repetition time=2110 milliseconds, 300 volumes over time, and slice order=ascending.

During resting-state fMRI data acquisition, participants will be asked to fixate on a cross displayed on the screen. At the end of this scan, participants must respond to written instructions by button press to check whether they were awake during the measurement.

**Figure 3:**
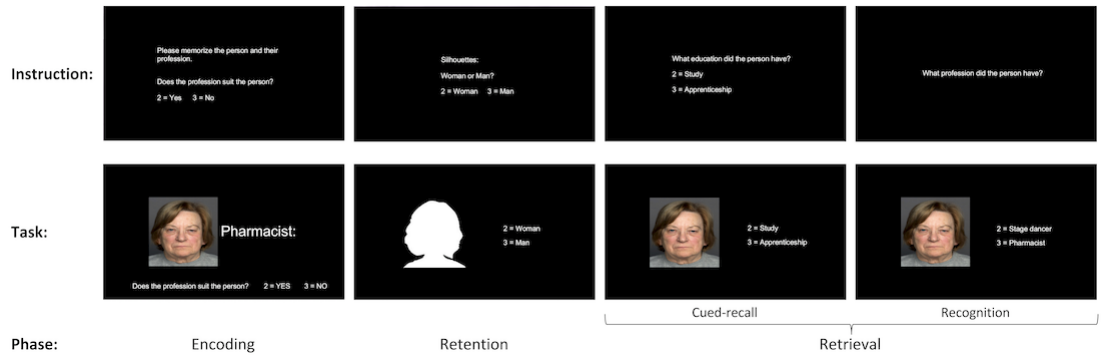
Graphical overview of the episodic memory task (face-occupation matching task).

In addition, task-based (event-related) fMRI measured with an episodic memory task (face-occupation matching task; duration: approximately 10 min) will be conducted. The face-occupation matching task was described in [140] and has been used in fMRI [141]. It was shown to robustly activate the hippocampus bilaterally and provide a differential signal for correct and incorrect trials [141]. The task was adapted specifically for older adults with mNCD [142] and will be run in PsychoPy (version 3.2.4) [143]. It includes 6 rounds, each containing (1) an encoding phase, (2) a retention phase, and (3) a retrieval phase including a cued recall task and a recognition task (see Figure 3 for an overview).

During the *encoding* phase, 5 face-occupation pairs will be presented in each round in a randomized order. A total of 30 face-occupation pairs (15 different face-occupation combinations) will be presented (Figure 3). Photographs of unfamiliar faces of older adults with neutral expressions were derived from the FACES database [144] and will be equally balanced over sex. Each face-occupation pair will be shown for 3.5 seconds. To ensure that participants view both faces and occupations, they will be asked to indicate whether each respective occupation “suits” (ie, pressing a button with their right index finger) or does not suit the presented face (ie, pressing a button with their right middle finger).

During the *retention* phase, 5 silhouettes will be presented in randomized order in each round. A total of 30 silhouettes will be presented. Each silhouette will be shown for 3.5 seconds. Participants will be asked to indicate by button press whether each silhouette corresponds to a woman (pressing a button with their right index finger) or a man (pressing a button with their right middle finger).

In the *retrieval* phase, a cued-recall task will be performed followed by a recognition task. In the cued recall task, participants will be asked to decide which of 2 educational degrees (ie, study [pressing a button with their right index finger] or apprenticeship [pressing a button with their right middle finger]) the previously encoded occupation of the person matched. In the recognition task, participants will have to decide between correct and distractor occupation via a button press (right index finger or right middle finger). For both the tasks, the response window will be 3.5 seconds. The photographs will be presented in the same order as those in the encoding phase.

The same recording parameters as in resting-state fMRI will be used. There are 4 alternative task conditions (ie, other face-occupation pairs). The task condition will be randomly allocated using random.org [145], while ensuring that 2 different conditions are used in the pre- and postintervention measurements for each participant.

##### Brain Structure and Function: Data Analysis

Processing and volumetric segmentation of *t*_1_w morphological data is performed using the longitudinal pipeline of the FreeSurfer software package (version 7.2.0 or newer with default parameters) [146,147], as described in [148]. Gray matter volumes and WMVs are determined for the following key regions of interest (ROIs): total brain (ie, total brain volume without ventricles [brainsegvolnotvent] from the aseg file), hippocampus, dorsolateral prefrontal cortex, PFC, and anterior cingulate cortex (ACC). Similar to Anderson-Hanley et al [77,78], we will combine the following regions to obtain the defined key ROIs of the dorsolateral prefrontal cortex, PFC, and ACC, because these are not directly extracted using the FreeSurfer software package: dlPFC=frontal middle gyrus and sulcus from the Destrieux et al [149] atlas; ACC=rostral and caudal anterior cingulate cortices from the Desikan et al [150] atlas; PFC=ACC+medial orbitofrontal and transverse frontopolar regions [151].

DTI data will be processed using the TractoFlow pipeline [152,153], a diffusion MRI tractography processing pipeline based on Nextflow [154] and Singularity [155], for human brain tractography reconstruction. In patients with m-MNCD, DTI abnormalities are concentrated in the posterior regions, whereas the most reported regions of DTI alterations are the temporal lobes, with a particular emphasis on the parahippocampal white matter and posterior cingulum [156,157]. Therefore, MD and FA will be calculated for the parahippocampal white matter and posterior cingulum, defined as key ROIs for this study. In addition, tract-based spatial statistical analysis will be performed to study white matter changes at voxel-to-voxel (whole-brain) levels. Tract-based spatial statistical analysis is based on a general linear model and will be performed using FSL’s randomization tool [158] with 5000 permutations to correct for multiple comparisons (*P*<.05, corrected). All results will include threshold-free cluster enhancement [159]. The threshold-free cluster enhancement correction method is somewhat similar to cluster-based thresholding but is generally more robust and avoids the need for an arbitrary initial cluster-forming threshold. Two contrasts will be computed at the individual and group levels, testing for positive and negative differences in FA and MD parameters pre- and postintervention. We will include age and sex as key covariates in the general linear model.

Data of *resting state and task-based fMRI* will be preprocessed to minimize data artifacts from thermal noise of MRI, system noise of the MR hardware, and subject-related noise resulting mostly from head motion [160]. The preprocessing steps will include slice time correction, motion correction, coregistration, and spatial smoothing (Gaussian kernel with 7 mm full width at half maximum) of the signal [161]. For functional connectivity (FC) analysis, preprocessed resting-state functional data will be analyzed using the latest release of the Functional Connectivity Toolbox (currently CONN 21a; [162,163]) in SPM12. The CONN utilizes a component-based noise correction method (CompCor) that increases selectivity and sensitivity and allows a higher degree of interscan reliability [164]. In addition to the described preprocessing steps, a band-pass filter (0.01-0.1 Hz) will be applied to remove linear drift artifacts and high-frequency noise. The CONN also accounts for outlier data points and movement time courses as nuisance regressors. The 6 motion parameters, WM and CSF, will be included as regressors of no interest, thereby reducing noise and signals that are unlikely to reflect neuronal activity related to FC. Age and sex will be included as key covariates of no interest. Significance was set at *P*<.05 with Family Wise Error-level correction for multiple comparisons (with *P*<.05 2-sided false-discovery rate correction) [163].

Bilateral hippocampal masks were selected as seeds from an Anatomical Automatic Labeling template [165]. Individual FC maps for the hippocampal seed will then be generated based on correlations between the mean signal time course within each seed region and the rest of the brain, similar to Suo et al 2016 [166], with the following selected key ROIs (ie, brain regions related to cognitive functioning, similar to Zhong et al [167]): precuneus or posterior cingulate cortex, medial prefrontal cortex, medial temporal lobe, angular gyrus, lateral temporal cortex, and medial, lateral, and inferior parietal cortex.

##### Spatiotemporal Parameters of Gait

Spatiotemporal parameters of gait will be evaluated to explore whether the addition of the “Brain-IT” training to usual care effectively improves gait. The gait of individuals with mNCD differs from that of healthy controls in terms of (among others; 1) slower single-task gait speed at the preferred walking speed (m/s) [18,168], (2) longer stride duration (ms) [18,168], (3) shorter stride length (cm) [18,168], (4) longer stance time (ms) [168], (5) longer swing time (ms) [168], (6) longer single support time (ms) [168], and (7) longer double support time (ms) [168]. Furthermore, individuals with mNCD typically show greater variability and coefficient of variation of gait parameters compared with cognitively healthy individuals [168].

According to the literature summarized above, we assume that an effective intervention leads to an (1) increase in single-task gait speed at the preferred walking speed, (2) decrease in stride duration, (3) increase in stride length, (4) decrease in stance time, (5) decrease in swing time, (6) decrease in single support time, and (7) decrease in double support time.

On the basis of this, we have elaborated our alternative hypotheses in more detail. It is hypothesized that in older adults with mNCD, the addition of the “Brain-IT” training to usual care results in differing effects on (c_1_) single-task gait speed at the preferred walking speed, (c_2_) stride duration, (c_3_) stride length, (c_4_) stance time, (c_5_) swing time, (c_6_) single support time, and (c_7_) double support time compared with usual care.

Spatiotemporal gait parameters will be assessed using a BTS G-WALK (BTS Bioengineering SpA, Garbagnate Milanese, Italy) inertial sensor attached with semielastic belt to participants’ lower back. The BTS G-WALK sensor delivers valid [169-171] and reliable [169,172] spatiotemporal gait parameters. All the acceleration data will be sampled at a frequency of 100 Hz. Data will be transmitted for analysis through Bluetooth 3.0, connection to the software program BTS G-Studio (BTS Bioengineering SpA, Italy). A gait-analysis protocol consisting of a figure of 8 walking path (ie, distance between cones of approximately 8 m) will be applied [108]. At least 50 consecutive gait cycles are required to ensure the reliability of the spatial and temporal parameters of gait variability [173]. Therefore, participants will perform 5 to 10 repetitions of the figure of 8 walking path at the preferred walking speed, depending on their walking speed and stride length. Comparative quantitative reference values for healthy older adults are available [174].

##### IADL Functioning

IADL functioning will be assessed using the Amsterdam IADL questionnaire short version, German for Switzerland, which has demonstrated good psychometric properties [109]. In addition, the original version of the Amsterdam IADL questionnaire was sensitive to longitudinal changes [175] and has been recommended for use in research settings [176]. The closest informant (eg, spouse, child, or friend) will fill out the questionnaire twice (within 2 weeks before the study participant starts or completes the intervention). Each item is scored on a 5-point Likert scale (“no difficulty” to “unable to perform”). Scoring is based on the item response theory. Item response theory latent trait levels are transformed to a T-score, with a range from 20 to 80, a mean of 50, and an SD of 10. A higher *t* score indicates better functioning [109].

##### Psychosocial Factors

QoL will be evaluated in interview format using the Quality of Life-Alzheimer Disease (QOL-AD) scale [110]. The QOL-AD is a valid and reliable self-report 13-item scale assessing various domains of QOL in cognitively impaired patients [110,177]. The German version of the QOL-AD scale, which has high test-retest reliability and good construct validity [111,112] will be used. Administration and evaluation will follow standardized instructions [112,178]. Comparable values are available for individuals with mNCD [179].

Levels of depression, anxiety and stress will be assessed using the short version of the Depression, Anxiety and Stress scale-21 (DASS-21) [113-115]. The DASS-21 has high reliability and good convergent and discriminant validity [113,180]. The validated German version of the DASS-21 will be administered and scored according to guidelines and scoring template [116,117]. Normative data of the 3 subscales are available and suggest cut-off scores of 10, 8, and 15, indicating significant depression, anxiety, or stress, respectively [116]. Comparative values for individuals with mNCD are available [181].

##### Cardiac Vagal Modulation (Resting Vagally-Mediated Heart Rate Variability)

To determine resting vm-HRV, all participants will be instructed to sit in a comfortable position on a chair without speaking, both feet flat on the floor with knees at a 90º angle, hands on the thighs (ie, palms facing upward), and eyes closed [95]. Measurements will be performed in a quiet room with dimmed light at room temperature. Data will be collected using a heart rate monitor (Polar M430) and sensor (Polar H10). The initial acclimatization phase will last for 5 minutes followed by a 5-minute resting measurement, the recommended standard duration for short-term recordings [95,182]. The start of the resting measurement will not be announced to the participants [95].

For resting HRV measurements, a sampling rate of 1000 Hz will be used to provide a temporal resolution of 1 milliseconds for each RR interval [183]. R-R data recordings will directly be transmitted to the Kubios HRV Premium (Kubios Oy, Kuopio, Finland, version 3.4) for analysis. Kubios HRV is a scientifically validated software for HRV analysis and has achieved the gold standard status in research [118,184-186]. The automatic beat correction algorithm and noise handling provided by the software will be used to correct for artifacts or ectopic beats. This algorithm has been validated for measurements at rest [184]. After removing interbeat-interval time series nonstationarities by detrending analysis using the smoothness priors method approach (settings: detrending method=smoothing priors, Lambda=500, f_c_=0.035 Hz), the mean values of mainly vagal-mediated HRV indices will be calculated for each segment. For this purpose, the mean R-R time intervals (mRR; ms), root mean square of successive RR interval differences (ms), percentage of successive RR intervals that differ by more than 50 milliseconds (%), absolute power of the high-frequency (0.15-0.4 Hz; high frequency [HF]) band (ms^2^), relative power of HF (in normal units; HF [n.u.] = HF [ms^2^] / (total power [ms^2^]—very low frequency; 0.00-0.04 Hz [ms^2^]), and Poincaré plot SD perpendicular to the line of identity (SD1; ms) will be considered [95,182,187-189]. In addition, the parasympathetic nervous system tone index (parasympathetic nervous system index) will be calculated to compare parasympathetic nervous system activity with normal resting values [189].

#### Other Endpoints

##### Safety Endpoint Variables

A protocol will be kept of all (serious) adverse events.

##### Baseline Factors

Baseline factors are collected through demographic data, including age, sex, height, weight, BMI, years of education, physical activity behavior (ie, measured with the German version of the International Physical Activity Questionnaire Short Form—short form [190,191] and analyzed according to published guidelines for data processing and analysis of International Physical Activity Questionnaire Short Form—short form [192]), clinical subtype (ie, mNCD due to AD, mild Frontotemporal NCD, mNCD with Lewy Bodies, or mild vascular NCD), medication intake, and changes in medication intake between pre- and postintervention measurements.

##### Adherence and Compliance Protocol

For the intervention group (ie, exergame training):

- Adherence Protocol (number of sessions completed per week per participant; automatically assessed in the Exergame Training Software) to calculate the mean adherence rate (%) = number of training sessions attended / total number of training sessions offered; calculated as the average of each participant’s weekly adherence with a maximum of 100%.
- Compliance Protocol (training time completed per week per participant; automatically assessed in the Exergame Training Software) to calculate the mean compliance rate (%) = training duration attended (min) / total training duration offered (min), calculated as the average of each participant’s weekly compliance, with a maximum of 100%.

### Participant Timeline

Table 3 provides an overview of the participant timeline.

**Table 3:** Participant timeline.

Time	≥2 days before premeasurements	≥1 day before premeasurements	Week−1 to 0	Week 1-12	Week 13-14	Week 15
Visit	Information	Consent and screening	Premeasurements	Intervention period	Postintervention measurements	Postintervention information
Location	At the interested persons’ home or at one of the study centers, depending on their preferences	At the interested persons’ home or at one of the study centers, depending on their preferences	At one of the study centers	At the participants’ homes	At one of the study centers	At the participants’ homes or at one of the study centers, depending on their preferences
Oral and written information	✓					
Written informed consent		✓				
Inclusion and exclusion criteria		✓				
Primary outcome:Qmci^a^ (~5 min)			✓		✓	
Secondary outcomes: WMS-IV-LM^b^ (~15 min) PEBL^c^-DSF^d^ and DSB^e^ (~5 min) PEBL-TMT-A and B^f^ (~5 min) TAP Go-NoGo^g^ (~5 min) HOTAP-A^h^ (~5 min) PEBL-MRT^i^ (~5 min) MRI^j^-Scan (~60 min) gait Analysis (~5 min) IADL^k^ (~15 min) QOL-AD^l^ (~5 min) DASS-21^m^ (~5 min) resting vm-HRV^n^ (~12 min)			✓		✓	
Baseline Factors (~5 min)			✓			
Safety endpoints			✓	✓	✓	
Adherence and Compliance to the Training Protocol				✓		
Study intervention				✓ (Study visits only applicable for exergame-group)		
Discuss individual results						✓

^a^Qmci: quick mild cognitive impairment screen.

^b^WMS-IV-LM: subtest “logical memory” of the Wechsler Memory Scale–fourth edition.

^c^PEBL: psychology experiment building language.

^d^DSF: digit span forward.

^e^DSB: digit span backward.

^f^TMT-A and B: Trail Making Test Part A and B.

^g^TAP Go-NoGo: subtest “Go-NoGo” of the test of attentional performance.

^h^HOTAP-A: HOTAP picture-sorting test part A.

^i^MRT: mental rotation task.

^j^MRI: magnetic resonance imaging

^k^IADL: instrumental activities of daily living.

^l^QOL-AD: Quality of Life-Alzheimer Disease.

^m^DASS-21: Depression, Anxiety and Stress scale-21.

^n^vm-HRV: vagally-mediated heart rate variability.

### Sample Size

The optimal sample size was justified based on Whitehead et al [193], and the following assumptions: the future main (full-scale) RCT will be planned with identical design and primary outcome as this study, with a two-sided type 1 error rate of 5% and a statistical power of 80%.

Our pilot feasibility RCT revealed a medium effect size (η^2^_p_=0.080; under peer review; registered at ClinicalTrials.gov NCT04996654). The observed medium effect size is slightly higher than the pooled evidence of exergame-based or combined motor-cognitive training interventions in older adults with NCD on global cognitive functioning. For exergame-based interventions in individuals with mNCD, Wang et al [194] reported a medium SMD of 0.57 (*P*=.21, 95% CI −0.32 to 1.47; *k*=1; n=20, compared with cognitively active control), while Gavelin et al (2021) reported an SMD of 0.13 (*P*>.05, 95% CI −0.22 to 0.48; *k*=2; n=109, compared with passive control) [63]. Stanmore et al [195] reported a small pooled effect of Hedges *g*=0.340 (*P*=.02, 95% CI 0.06-0.62; *k*=6; n=193) for patients with cognitive impairment. Further meta-analytic results for individuals with mNCD are limited to simultaneous motor-cognitive training with reported small-to-medium effect sizes, including SMDs of 0.45 [61], 0.48 [196], 0.531 [61], and 0.69 [197]. On the basis of this, a medium effect size of a SMD of 0.5 seems reasonable and is anticipated. This leads to a justified optimal sample size of n=14 per arm.

In our pilot feasibility RCT, we observed an attrition rate of 20% (under peer review, registered at ClinicalTrials.gov NCT04996654). This is consistent with recent systematic reviews synthesizing mean attrition rates of 17% (range, 0 to 59%) [198] in physical training interventions, 10% for combined motor-cognitive training interventions [199], and 15% (range 0%-31%) [200] for exergame-based interventions for patients with m-MNCD. Therefore, an attrition rate of 20% is anticipated. To ensure an adequate number of participants, a wide upper safety margin for an attrition rate of up to 40% is selected.

On the basis of these considerations, we will aim to recruit 17 to 20 older adults with mNCD per group, leading to a total sample size of 34 to 40. This will provide a sufficiently precise estimate of the treatment effect to minimize the sample size needed for a future full-scale RCT [193].

### Randomization

#### Sequence Generation

Participants will be randomly allocated to the intervention or control group. Variable block randomization (ie, block sizes=4, 6, and 8) with a 1:1 allocation ratio stratified by sex and institute (study center) will be used.

#### Allocation Concealment Mechanism

To ensure allocation concealment, random allocation will be computer generated using a validated variable block randomization model implemented in the data management system Castor EDC (Ciwit BV) [201].

#### Implementation

Randomization process will be set up by PM before starting patient recruitment. PM will also oversee the enrollment of participants. Participants will individually be assigned to the intervention or control group by the investigator responsible for supervision and correspondence with the respective study participant after completing premeasurements. Randomization allocation can be viewed in eCRFs by investigators who have been assigned right to do so. The eCRF is implemented in the Castor EDC data management system [201]. None of the investigators performing postintervention measurements will have access rights to view the randomization allocation in eRCFs.

### Blinding

Outcome evaluators of pre- and postintervention measurements will be blinded to the group allocation (single blinding). To ensure blinding and blind-keeping of outcome assessors, detailed study-specific guidelines for all relevant procedures related to blinding and blind-keeping of outcome assessors have been established, which will be followed by all involved study investigators. For data assessed throughout the intervention period (ie, only applicable to the intervention group), blinding of investigators is not possible. The blinding of participants will also not be possible because usual care will be used as a control intervention.

### Participant Retention

Once a patient is included, a trained investigator will be assigned as the person responsible for supervision and correspondence with the respective study participant and will make all reasonable efforts to achieve participant’s retention in the study. Examples include providing written information sheets and reminders about study appointments, involving carers or relatives as personal support for study participants, and providing assistance with travel to the study center. Specifically, in the intervention group, each participant is provided with a training manual that is individually adapted to ensure that they use the training system correctly.

### Data Management

All study investigators will be thoroughly trained for all study procedures according to the Guidelines of Good Clinical Practice and in line with detailed working instructions and a data management plan (Multimedia Appendix 1). In short, local principal investigators are in charge for methodological standards and quality of data collection using data management system Castor EDC (Ciwit BV) [201]. The data management plan specifies standard procedures for data management, evaluation, and storage. All data entries will be cross-checked by a second investigator before exporting for analysis. Range checks for data values were preprogrammed for data entry into the eCRFs. To minimize bias during the assessment of all clinical outcome measures, detailed working instructions were prepared, including standardized measurement procedures and standardized instructions of participants for all measurements.

### Statistical Methods

Statistical analysis will be executed using R (The R Foundation), in line with RStudio (RStudio Inc). For demographics as well as training adherence and compliance, all collected data will be included (ie, including data of dropouts up to the time point of their withdrawal). For primary and all secondary outcomes, the data of all participants who completed pre- and postintervention measurements, regardless of protocol adherence, will be included in the statistical analyses. Questionnaire scores will be regarded as ordinal data. Data will be reported as mean (SD) values for continuous parametric data and median (IQR) values for continuous nonparametric data.

For all outcomes, descriptive statistics will be computed first. Normality distribution of data will be checked using the Shapiro-Wilk test. The level of significance will be set to *P*≤.05 (2-sided, uncorrected).

For all demographic variables, between-group differences (ie, intervention vs control) will be tested using an independent *t* test or Mann-Whitney *U* test in case the data are not normally distributed. For primary and secondary outcomes, the assumption of homogeneity of variance will be checked using Levene test. In case all assumptions for a 2-way analysis of covariance (ANCOVA) are met, effectiveness of the “Brain-IT” training will be analyzed using a two-way ANCOVA with the premeasurement value as covariate for the predicting group factor and the postmeasurement value as outcome variable [202]. If not all assumptions are met, the Quade nonparametric ANCOVA will be used. Because of the stratification by sex in the randomization process, no sex-specific statistical analysis will be computed. However, if there are relevant between-group differences in sex distribution or other demographic variables, these will be included in the statistical analyses as additional covariates. To determine whether the effects are substantive, partial eta-squared (η^2^_p_) effect sizes will be calculated for all primary and secondary outcomes. Effect sizes will be interpreted as small (0.01≤η^2^_p_<0.06), medium (0.06≤η^2^_p_<0.14), or large (η^2^_p_>0.14) [203].

Statistical analysis will be performed by PM after data collection is completed. As this is a small-scale RCT, no interim analysis will be performed.

### Monitoring

External monitoring will be performed at three time points: (1) before starting recruitment (site initiation visit); (2) after inclusion of 10 participants (routine monitoring visit); and (3) after completing all postintervention measurements and database lock (close-out visit) by Dr Ruud Knols (Physiotherapy and Occupational Therapy Research Centre, Directorate of Research and Education, University Hospital Zurich). Monitoring activities will be performed according to the ICH-GCP and according to a detailed monitoring plan that is based on adaptations of the “Monitoring Plan Template” of the Swiss Clinical Trial Organization to meet study-specific requirements.

### Ethics and Dissemination

#### Research Ethics Approval and Protocol Amendments

The study protocol was approved by the Ethics Committees of Zurich and Eastern Switzerland (EK-2022-00386). Any substantial amendment to the study protocol will have to be approved by the Ethics Committees of Zurich and Eastern Switzerland, and the trial registration at clinicaltrials.gov (NCT05387057) will be updated accordingly. Any substantial amendments to the study protocol that may occur after publication of this study protocol will be reported in the final publication of the study results.

#### Consent or Assent

As described in detail in section “Methods - Recruitment“, suitable patients willing to take part in the study will provide written informed consent in a second in-person meeting with one of the trained investigators of the study team at the interested persons home or at one of the study centers, depending on their preferences.

#### Confidentiality

Measures to ensure data confidentiality are detailed in the data management plan (Multimedia Appendix 1).

#### Access to Data

As detailed in the data management plan (Multimedia Appendix 1), anonymized data sets from this project that underpin a publication will be deposited in the Zenodo repository and made public after completing data collection.

#### Ancillary and Posttrial Care

In the event of study-related damage or injuries, the liability of the institution ETH Zurich provides compensation, except for claims arising from misconduct or gross negligence. Insurance is covered by ETH Zurich public liability insurance (Police Nr 30/4.078.362 of “Basler Versicherung AG”). After completion of study participation, all participants will be offered to use the training device at their own responsibility, either by purchasing a “Senso Flex” (if market launch of the “Senso Flex” has already taken place at that time) or by using a “Senso” in one of a nearby physiotherapies, nursing homes or rehabilitation clinics offering the system.

#### Dissemination Policy

Each participant will be informed of their personal results by providing a written report summarizing the patients’ relevant results. The report will be handed out in person and explained by a trained investigator to ensure that the patients understand their results. In addition, upon request, an information letter will be sent to participants at the end of the study to inform them about the findings obtained in this study.

A manuscript of the results will be published in a peer-review open-access journal as well as on ResearchGate and original data sets will be made available in a publicly accessible repository. Standard journal authorship criteria will be applied; there will be no use of professional writers. In addition, the results of this study will be disseminated via open-access journal articles and conference presentations to inform health care professionals, the public, and other relevant groups about the study results and the knowledge gained.

## Results

Upon the initial submission of this study protocol, 13 patients were contacted by the study team. Four patients were included in the study, 2 were excluded because they were not eligible, and 7 were being informed about the study in detail. Of the 4 included patients, 2 already completed all premeasurements and were in week 2 of the intervention period. Data collection is expected to be completed by December 2023. A manuscript of the results will be submitted for publication in a peer-reviewed open-access journal in 2024.

## Discussion

### Principal Findings

This RCT will systematically evaluate the effectiveness of a newly developed nonpharmacological exergame-based training targeted at improving cognitive functioning in older adults with mNCD. The results of this study should provide information on whether the addition of our modified and improved exergame-based training (according to the “Brain-IT” training concept) to usual care is effective in improving global cognitive functioning, and a future full-scale RCT is warranted. The study will thus contribute to the evidence base in prevention of disability because of cognitive impairment, which has been declared a public health priority by the World Health Organization [33].

### Comparison With Prior Work

Technological innovations (eg, exergames) provide new options to engage older adults with mNCD in (simultaneously-incorporated) motor-cognitive training [70]. So far, only 1 study has applied exergame-based training that was individualized content-wise based on the cognitive abilities of older adults with mNCD [82]. Furthermore, most previous studies used commercially available exergame systems [76-82], where the training content was not specifically developed for individuals with mNCD. These studies have shown rather limited effects on global cognitive functioning [63], although combined motor-cognitive training seems to be the most effective type of training for improving cognition in older adults with mNCD [61,63-65].

### Limitations

There are some limitations to this RCT that must be mentioned. First, we will only explore the effectiveness of the addition of the “Brain-IT” training concept to usual care. In line with this, the sample size for this RCT was justified to provide a sufficiently precise estimate of the treatment effect to minimize the sample size needed for a future full-scale RCT [193]. As a result, the statistical analyses will most probably be insufficiently powered, and confirmatory studies are needed following this study. Second, usual care interventions are assessed by self-report of patients. To counteract possible biased information, the study team will ask specific questions about whether participants are engaged in typical usual care interventions (as described in section “the Methods and Materials section—Interventions—Control Group”) and actively involve participants’ proxies when collecting this information. Third, patients screened for MCI according to predefined criteria are recruited in addition to patients with a clinical diagnosis of mNCD, which will increase the heterogeneity of the study population. In line with this, in our project, we aim to investigate an individualized exergame-based training concept not only to treat clinically diagnosed patients with mNCD but also to prevent progression to dementia in individuals at risk who might not have been diagnosed (yet).

### Conclusions

This study will contribute to the evidence base in the highly relevant area of prevention of disability because of cognitive impairment, which has been declared a public health priority by the World Health Organization.

## Appendix

Multimedia Appendix 1 Standard Protocol Items: Recommendations for Interventional Trials (SPIRIT) 2013 checklist and data management plan.

## Abbreviations

ACC: anterior cingulate cortex
AD: Alzheimer disease
ADAS-cog: Alzheimer’s Disease Assessment Scale-Cognitive Subscale
ANCOVA: analysis of covariance
CDIP: Canadian Dementia Imaging Protocol
DASS-21: Depression, Anxiety and Stress Scale-21
DSF: digit span forward
DSM-V: Diagnostic and Statistical Manual of Mental Disorders 5th Edition
DTI: diffusion tensor imaging
FC: functional connectivity
HF: high frequency
IADL: instrumental activities of daily living
MCI: mild cognitive impairment
MNCD: major neurocognitive disorder
mNCD: mild neurocognitive disorder
MRI: magnetic resonance imaging
PEBL: psychology experiment building language
Qmci: quick mild cognitive impairment
RCT: randomized controlled trial
ROI: region of interest
SMD: standardized mean difference
TAP: test of attentional performance
