# Supplementary material for: Effectiveness of an Individualized Exergame-Based Motor-Cognitive Training Concept Targeted to Improve Cognitive Functioning in Older Adults With Mild Neurocognitive Disorder: Study Protocol for a Randomized Controlled Trial
Source: JMIR Res Protoc. 2023 Feb 6;12:e41173. doi: 10.2196/41173 (PMC9941909; doi:10.2196/41173)
Supplement: Multimedia Appendix 1 [file resprot_v12i1e41173_app1.pdf]

Supplementary Files

–

Effectiveness of an Individualized Exergame-Based Motor-Cognitive Training Concept Targeted to Improve Cognitive Functioning in Older Adults with Mild Neurocognitive Disorder:

Study Protocol for a Randomized Controlled Trial

Supplementary File 1

–

SPIRIT 2013 Checklist

SPIRIT
STANDARD PROTOCOL ITEMS: RECOMMENDATIONS FOR INTERVENTIONAL TRIALS

Table 1: SPIRIT 2013 Checklist: Recommended items to address in a clinical trial protocol and related documents* [1, 2]

Section/item	Item No	Description	Reported in section
Administrative information			
Title	1	Descriptive title identifying the study design, population, interventions, and, if applicable, trial acronym	Title page
Trial registration	2a	Trial identifier and registry name. If not yet registered, name of intended registry	Methods - Trial Design and Study Setting
	2b	All items from the World Health Organization Trial Registration Data Set	Methods - Trial Design and Study Setting (Table 1)
Protocol version	3	Date and version identifier	Methods
Funding	4	Sources and types of financial, material, and other support	Discussion - Acknowledgments
Roles and responsibilities	5a	Names, affiliations, and roles of protocol contributors	Discussion - Authors' Contributions
	5b	Name and contact information for the trial sponsor	Methods - Trial Design and Study Setting (Table 1)
	5c	Role of study sponsor and funders, if any, in study design; collection, management, analysis, and interpretation of data; writing of the report; and the decision to submit the report for publication, including whether they will have ultimate authority over any of these activities	Discussion - Acknowledgments
	5d	Composition, roles, and responsibilities of the coordinating center, steering committee, endpoint adjudication committee, data management team, and other individuals or groups overseeing the trial, if applicable (see Item 21a for data monitoring committee)	N/A
Introduction			
Background and rationale	6a	Description of research question and justification for undertaking the trial, including summary of relevant studies (published and unpublished) examining benefits and harms for each intervention	Introduction - Background
	6b	Explanation for choice of comparators	Introduction - Background
Objectives	7	Specific objectives or hypotheses	Introduction – Objectives and Hypotheses
Trial design	8	Description of trial design including type of trial (eg, parallel group, crossover, factorial, single group), allocation ratio, and framework (eg, superiority, equivalence, noninferiority, exploratory)	Methods – Trial Design and Study Setting

Methods: Participants, interventions, and outcomes

Study setting	9	Description of study settings (eg, community clinic, academic hospital) and list of countries where data will be collected. Reference to where list of study sites can be obtained	Methods – Trial Design and Study Setting
Eligibility criteria	10	Inclusion and exclusion criteria for participants. If applicable, eligibility criteria for study centers and individuals who will perform the interventions (eg, surgeons, psychotherapists)	Methods – Eligibility Criteria
Interventions	11a	Interventions for each group with sufficient detail to allow replication, including how and when they will be administered	Methods - Interventions
	11b	Criteria for discontinuing or modifying allocated interventions for a given trial participant (eg, drug dose change in response to harms, participant request, or improving/worsening disease)	Methods - Interventions
	11c	Strategies to improve adherence to intervention protocols, and any procedures for monitoring adherence (eg, drug tablet return, laboratory tests)	Methods – Interventions, Methods - Outcomes – Other Endpoints - Adherence and Compliance Protocol
	11d	Relevant concomitant care and interventions that are permitted or prohibited during the trial	Methods – Interventions
Outcomes	12	Primary, secondary, and other outcomes, including the specific measurement variable (eg, systolic blood pressure), analysis metric (eg, change from baseline, final value, time to event), method of aggregation (eg, median, proportion), and time point for each outcome. Explanation of the clinical relevance of chosen efficacy and harm outcomes is strongly recommended	Methods - Outcomes
Participant timeline	13	Time schedule of enrolment, interventions (including any run-ins and washouts), assessments, and visits for participants. A schematic diagram is highly recommended (see Figure)	Methods – Participant Timeline
Sample size	14	Estimated number of participants needed to achieve study objectives and how it was determined, including clinical and statistical assumptions supporting any sample size calculations	Methods – Sample Size
Recruitment	15	Strategies for achieving adequate participant enrolment to reach target sample size	Methods - Recruitment

Methods: Assignment of interventions (for controlled trials)

Allocation:

Sequence generation	16a	Method of generating the allocation sequence (eg, computer-generated random numbers), and list of any factors for stratification. To reduce predictability of a random sequence, details of any planned restriction (eg, blocking) should be provided in a separate document that is unavailable to those who enrol participants or assign interventions	Methods – Randomization – Sequence Generation
Allocation concealment mechanism	16b	Mechanism of implementing the allocation sequence (eg, central telephone; sequentially numbered, opaque, sealed envelopes), describing any steps to conceal the sequence until interventions are assigned	Methods – Randomization – Allocation Concealment Mechanism
Implementation	16c	Who will generate the allocation sequence, who will enroll participants, and who will assign participants to interventions	Methods – Randomization – Implementation
Blinding (masking)	17a	Who will be blinded after assignment to interventions (eg, trial participants, care providers, outcome assessors, data analysts), and how	Methods – Blinding
	17b	If blinded, circumstances under which unblinding is permissible, and procedure for revealing a participant's allocated intervention during the trial	N/A (no unblinding)

Methods: Data collection, management, and analysis

Data collection methods	18a	Plans for assessment and collection of outcome, baseline, and other trial data, including any related processes to promote data quality (eg, duplicate measurements, training of assessors) and a description of study instruments (eg, questionnaires, laboratory tests) along with their reliability and validity, if known. Reference to where data collection forms can be found, if not in the protocol	Methods – Outcomes, Methods – Data Management

	18b	Plans to promote participant retention and complete follow-up, including list of any outcome data to be collected for participants who discontinue or deviate from intervention protocols	Methods - Participant Retention
Data management	19	Plans for data entry, coding, security, and storage, including any related processes to promote data quality (eg, double data entry; range checks for data values). Reference to where details of data management procedures can be found, if not in the protocol	Methods – Data Management
Statistical methods	20a	Statistical methods for analyzing primary and secondary outcomes. Reference to where other details of the statistical analysis plan can be found, if not in the protocol	Methods – Statistical Methods
	20b	Methods for any additional analyses (eg, subgroup and adjusted analyses)	Methods – Statistical Methods
	20c	Definition of analysis population relating to protocol non-adherence (eg, as randomized analysis), and any statistical methods to handle missing data (eg, multiple imputation)	Methods – Statistical Methods

<u>Methods: Monitoring</u>

Data monitoring	21a	Composition of data monitoring committee (DMC); summary of its role and reporting structure; statement of whether it is independent from the sponsor and competing interests; and reference to where further details about its charter can be found, if not in the protocol. Alternatively, an explanation of why a DMC is not needed	Methods – Monitoring
	21b	Description of any interim analyses and stopping guidelines, including who will have access to these interim results and make the final decision to terminate the trial	Methods – Statistical Methods
Harms	22	Plans for collecting, assessing, reporting, and managing solicited and spontaneously reported adverse events and other unintended effects of trial interventions or trial conduct	Methods - Outcomes – Other Endpoints - Safety Endpoint Variables
Auditing	23	Frequency and procedures for auditing trial conduct, if any, and whether the process will be independent from investigators and the sponsor	N/A

<u>Ethics and dissemination:</u>

Research ethics approval	24	Plans for seeking research ethics committee/institutional review board (REC/IRB) approval	Methods – Ethics Approval and Ethics and Methods – Ethics and Dissemination – Research Ethics Approval and Protocol Amendments
Protocol amendments	25	Plans for communicating important protocol modifications (eg, changes to eligibility criteria, outcomes, analyses) to relevant parties (eg, investigators, REC/IRBs, trial participants, trial registries, journals, regulators)	Methods – Ethics and Dissemination – Research Ethics Approval and Protocol Amendments
Consent or assent	26a	Who will obtain informed consent or assent from potential trial participants or authorized surrogates, and how (see Item 32)	Methods – Ethics and Dissemination – Consent or Assent
	26b	Additional consent provisions for collection and use of participant data and biological specimens in ancillary studies, if applicable	N/A
Confidentiality	27	How personal information about potential and enrolled participants will be collected, shared, and maintained in order to protect confidentiality before, during, and after the trial	Methods – Ethics and Dissemination – Confidentiality
Declaration of interests	28	Financial and other competing interests for principal investigators for the overall trial and each study site	Discussion - Conflict of Interest
Access to data	29	Statement of who will have access to the final trial dataset, and disclosure of contractual agreements that limit such access for investigators	Methods – Ethics and Dissemination – Access to Data
Ancillary and post-trial care	30	Provisions, if any, for ancillary and post-trial care, and for compensation to those who suffer harm from trial participation	Methods – Ethics and Dissemination – Ancillary and Post-Trial Care
Dissemination policy	31a	Plans for investigators and sponsor to communicate trial results to participants, healthcare professionals, the public, and other relevant groups (eg, via publication, reporting in results databases, or other data sharing arrangements), including any publication restrictions	Methods – Ethics and Dissemination – Dissemination Policy
	31b	Authorship eligibility guidelines and any intended use of professional writers	Methods – Ethics and Dissemination – Dissemination Policy

	31c	Plans, if any, for granting public access to the full protocol, participant-level dataset, and statistical code	Methods – Ethics and Dissemination – Dissemination Policy
<u>Appendices</u>			
Informed consent materials	32	Model consent form and other related documentation given to participants and authorized surrogates	All documents handed over to study participants have been approved by the ethics commission. They will be made available upon reasonable request.
Biological specimens	33	Plans for collection, laboratory evaluation, and storage of biological specimens for genetic or molecular analysis in the current trial and for future use in ancillary studies, if applicable	N/A

*It is strongly recommended that this checklist be read in conjunction with the SPIRIT 2013 Explanation & Elaboration for important clarification on the items. Amendments to the protocol should be tracked and dated. The SPIRIT checklist is copyrighted by the SPIRIT Group under the Creative Commons "Attribution-Non-Commercial-NoDerivs 3.0 Unported" license. [1, 2]

Supplementary File 2

–

Data Management Plan for Study ID = 2022-00386

Institutions:

Institute of Human Movement Sciences and Sport, ETH Zurich, Zurich, Switzerland;
AND
Department of Health, OST - Eastern Switzerland University of Applied Sciences, St. Gallen, Switzerland

Responsibilities and Data Management Contact Person:

Eling D. de Bruin (PI)[1,2,3] (eling.debruin@ost.ch or eling.debruin@hest.ethz.ch)
AND
Patrick Manser (delegated by PI as responsible data management person)[2] (patrick.manser@hest.ethz.ch)

[1]Department of Health, OST – Eastern Switzerland University of Applied Sciences, St. Gallen, Switzerland;
[2]Motor Control and Learning Group - Institute of Human Movement Sciences and Sport, Department of Health Sciences and Technology, ETH Zurich, Zurich, Switzerland;
[3]Division of Physiotherapy, Department of Neurobiology, Karolinska Institute, Stockholm, Sweden

1. Data collection and documentation

What data will you collect, observe, generate or re-use?

In this research project, the following quantitative data is recorded directly on the electronic case report forms (eCRF), in a separate source document (for all study centers data is locally stored on a secure ETH server of the Institute of Human Movement Sciences and Sport (ETH Zurich) in the following folder: smb://hest.nas.ethz.ch/green_groups_btl_public/Research Projects & Group/Patrick Manser_DATA/3 RCT/3_Data & Statistics/2_Data), or in paper format (e.g. questionnaires) in the trial master files. Selected data (all data collected except MRI data and data of the adherence and compliance protocol for the exergame group) will be transmitted to the eCRFs and will be exported for statistical analysis as CSV files. This includes the following:

- Quantitative Data of 18 clinical outcome measures including 42 outcome variables of up to 40 study participants assessed at the Pre-, and Post-Measurements, including:
    - Total point score [] of the Quick Mild Cognitive Impairment Screen (Qmci); evaluated on separate source document (printed questionnaire) and transferred to the eCRF
    - Total point scores [] of part 1, part 2 and the recognition part of the subtests 'logical memory' of the Wechsler Memory Scale-Revised (WMS-IV-LM); evaluated on separate source document (printed questionnaire) and transferred to the eCRF
    - Total point score [] and maximum span [] of the computerized version of the Digit Span Forward test (PEBL-DSF); txt.-file of data that is transferred to the eCRF
    - Completion time [s] and number of errors [] of the computerized version of the Trail Making Test – Part A (PEBL-TMT-A); csv.-file of data that is transferred to the eCRF

- Median reaction time [ms] and number of errors [] of the subtest 'Go-NoGo' of the Test of Attentional Performance (TAP Go-NoGo); rtf.-file of data that is transferred to the eCRF
- Combi score (i.e. sum of the points [] divided by the time they needed to arrange the cards [min]) [points · min^{-1}] of the HOTAP picture-sorting test part A (HOTAP-A); evaluated on separate source document (printed questionnaire) and transferred to the eCRF
- Total point score [] and maximum span [] of the computerized version of the Digit Span Backward (PEBL-DSB) test; txt.-file of data that is transferred to the eCRF
- Completion time [s] and number of errors [] of the computerized version of the Trail Making Test – Part B (PEBL-TMT-B); csv.-file of data that is transferred to the eCRF
- Median reaction time [ms] and performance (number of correct answered trials) [] of the computerized version of the Mental Rotation Task (PEBL MRT); csv.-file of data that is transferred to the eCRF
- Raw data files (approximately 600 megabyte per measurement) from MRI scans, nii.-, par.-, and rec.-files of raw data that is used for separate analysis as described in the study protocol
- Seven spatiotemporal parameters of gait (including: walking speed [m · s^{-1}], stride duration [ms], stride length [cm], stance phase duration [ms], swing time [ms], single support time [ms], and double support time [ms]) measured by portable inertial sensor and a figure-8 walking path; pdf.-file of computed data that is transferred to the eCRF
- T-Score of the Amsterdam IADL Questionnaire []; evaluated on separate source document (electronic questionnaire available on qualtrics^XM platform or printed questionnaire) and transferred to the eCRF
- Total score [] of the Quality of Life-Alzheimer's Disease (QOL-AD) scale; evaluated on separate source document (printed questionnaire) and transferred to the eCRF
- Composite scores [] of the Depression, Anxiety and Stress Scale-21 (DASS-21); evaluated on separate source document (printed questionnaire) and transferred to the eCRF
- Seven outcome variables for resting state vagally-mediated heart rate variability data derived from R-R Intervals (including: mean R-R time interval (mRR) [ms], root mean square of successive RR interval differences (RMSSD) [ms], the percentage of successive RR intervals that differ by more than 50 ms (pNN50) [%], the absolute power of the high-frequency (0.15 – 0.4 Hz; HF) band [ms^2], the relative power of HF (in normal units; HF [n.u.] = HF [ms^2] / (total power [ms^2] – very low frequency (0.00 – 0.04 Hz [ms^2])), and the Poincaré plot standard deviation perpendicular to the line of identity (SD1) [ms], and the parasympathetic nervous system tone index (PNS-Index) []; csv.file of data is used for HRV analysis (in Kubios HRV Premium (Kubios Oy, Kuopio, Finland, version 3.4)) of which data is transferred to the eCRF

- Quantitative Data of demographic and clinical characteristics of up to 40 study participants assessed at the pre- (and post-) measurements, including:
  - age [years]; data directly entered to the eCRF
  - sex [male, female]; data directly entered to the eCRF
  - height [m]; data directly entered to the eCRF
  - weight [kg]; data directly entered to the eCRF
  - body mass index (BMI) [kg · m^{-2}]; data automatically calculated by the data management system in the eCRF
  - years of education [years]; data directly entered to the eCRF
  - clinical subtype (i.e., mNCD due to Alzheimer's Disease, mild Frontotemporal NCD, mNCD with Lewy Bodies, or mild vascular NCD); data directly entered to the eCRF
  - medication intake (assessed both at PRE- and POST-Measurement; data directly entered to the eCRF

- Descriptive data about the Safety Protocol (number, type and detailed description (according to the guidelines of good clinical practice) of all (serious) adverse events); data directly entered to the eCRF

Additionally, quantitative data of the following additional endpoints will be recorded in separate source documents, and stored as csv. files:

- Adherence Protocol, to calculate:

- mean adherence rate [%] = number of training sessions attended / total number of training sessions offered; calculated as the average of each participants weekly adherence with a maximum of 100 %.; data entered into specific source file
- mean compliance rate [%] = training duration attended [min] / total training duration offered [min]); calculated as the average of each participants weekly compliance with a maximum of 100 %.; data entered into specific source file

How will the data be collected, observed or generated?

Data collection of all clinical outcome measures as well as the demographic and clinical characteristics of the study participants will be done by outcome assessors who will be thoroughly trained by the local principal investigators in using the measurement technologies and protocols as well as data management according to the Guidelines of Good Clinical Practice and considering detailed working instructions. The local principal investigators will be in charge for the methodological standards and quality of the data collection. To ensure that the outcome assessor of all clinical outcome measures is not influenced by the group allocation of the measured participant, the outcome evaluator of the pre-, and post-measurements will be blinded to group allocation. To minimize bias during the assessment of all clinical outcome measures, detailed working instructions were prepared that include standardized procedures and instructions of participants for all measurements as well as standardized procedures of data management, evaluation, and storage (including standardized naming conventions for the structure of folders and file names to organize the data). Whenever possible, validated measurement protocols were selected for this study and (if available) will be administered according to standardized protocols of developers/manufacturers. Data of all clinical outcome measures as well as the demographic and clinical characteristics will be recorded directly on the electronic case report forms (eCRF) or in a separate source document and on the electronic case report forms. The project leader will be in charge of setting up the eCRF using the data management system Castor EDC (Ciwit BV, Amsterdam, The Netherlands) [3]. The investigators will create eCRFs, one for each enrolled study participant, to be filled in with all relevant data pertaining to the subject during the study. The eCRF will only contain coded identification so that participants are not identified on the document by name or birth date and to ensure subject confidentiality. Appropriate coded identification will be used (i.e. an eight-digit random number will be generated by the data management system Castor EDC (Ciwit BV, Amsterdam, The Netherlands) [3] to encode each participant within the eCRF. It will be assured that any authorized person, who may perform data entries and changes in the eCRF, can be identified. All changes within the eCRF will automatically be reported in an audit trial by the data management system Castor EDC (Ciwit BV, Amsterdam, The Netherlands) [3]. The Audit Trial shows all changes that are made to the study, including changes both during building the form and during data entry. The investigators assure to perform a complete and accurate documentation of the participants' data in the eCRF. During the study, the eCRFs will be kept updated to always reflect the current participant status. Additionally, all data entries will be cross-checked by a second study investigator prior to export for analysis and range checks for data values were pre-programmed for data entry in the eCRF. All generated data will always be stored twice/double at the investigational sites (Institute of Human Movement Science and Sports, ETH Zurich or Department of Health, Eastern Switzerland University of Applied Sciences) in pseudonymized form on a secure network folder.

Data collection of all additional endpoints (i.e. adherence protocol, implementation evaluation, and cost effectiveness) will be done the following: The quantitative data on the adherence protocol will be collected by the responsible persons for supervision and correspondence with study participants using training logs and forms, including the number and duration of (completed) training sessions. All generated data will always be stored twice/double at the investigational sites (Institute of Human Movement Science and Sports, ETH Zurich or Department of Health, Eastern Switzerland University of Applied Sciences) in pseudonymized form on a secure network folder.

What documentation and metadata will you provide with the data?

For each dataset, a README file will be provided, including the name and persistent identifier of each dataset file, the date and time of data collection, and the names of the persons who collected the data. In addition, the data processing methods, analytical steps, and person identifier for persons involved in data processing or analytic steps, and assessments, if applicable, will be provided. The conditions to access the data will also be provided, such as a creative commons license.

2. Ethics, legal and security issues

How will ethical issues be addressed and handled?

Personal data and health-related data are sensitive personal data by the Swiss Federal Act on Data Protection. All trial and participant data will be handled with uttermost discretion and will only be accessible to authorized personnel who require the data to fulfil their duties within the scope of the study. All information of participants obtained because of this study will be considered confidential and disclosure to third parties is prohibited. All the investigators of the study are bound to professional discretion. Furthermore, all procedures must comply with the current Good Clinical Practice standards, the Declaration of Helsinki and the Swiss Human Research Act.

All potential subjects will be fully informed about the study procedures by providing verbal explanations and an information sheet. The purpose of the study as well as expected effects, benefits and risks of the study will be explained by the investigators, who will also be available to answer open questions and clarify uncertainties of participants. It will further be verified, that withdrawal is permitted at any time during the study without having to give a reason, highlighting that participation in the study is entirely voluntary. The study information sheet will include detailed information on data collection, storage, processing, and sharing/publication and will inform about a participants' right on information about the collected data as well as data deletion and data correction.

Personal data of participants will be encoded before recording, which makes it impossible to conclude on the individual research data without the subject master file (including the pseudonymization codes) and the randomization log. Only the leading project manager and the local principle investigators will be assigned the rights

to view the randomization log within the eCRF (data management system Castor EDC (Ciwit BV, Amsterdam, The Netherlands) [3]).

The investigators will create a record in the electronic case report forms (eCRF) using the data management system Castor EDC (Ciwit BV, Amsterdam, The Netherlands) [3], one for each enrolled study participant, to be filled in with all relevant data pertaining to the subject during the study. The eCRF will only contain coded identification so that participants are not identified on the document by name or birth date and to ensure subject confidentiality. Appropriate coded identification will be used (i.e. an eight-digit random number will be generated by the data management system Castor EDC (Ciwit BV, Amsterdam, The Netherlands) [3] to encode each participant within the eCRF. It will be assured that any authorized person, who may perform data entries and changes in the eCRF, can be identified. All changes within the eCRF will automatically be reported in an audit trial by the data management system Castor EDC (Ciwit BV, Amsterdam, The Netherlands) [3]. The Audit Trial shows all changes that are made to the study, including changes both during building the form and during data entry. The investigators assure to perform a complete and accurate documentation of the participants' data in the eCRF. During the study, the eCRFs will be kept updated to always reflect the current participant status. All data entries will be cross-checked by a second study investigator prior to export for analysis. Exported data will still be encoded using the coded identification and will always be stored twice/double at the investigational sites (Institute of Human Movement Science and Sports, ETH Zurich or Department of Health, Eastern Switzerland University of Applied Sciences) on a secure network folder. Trial and participant data will be handled with uttermost discretion and will only be accessible to authorized personnel who require the data to fulfil their duties within the scope of the study. All information of participants obtained because of this study will be considered confidential and disclosure to third parties is prohibited. All the investigators of the study are bound to professional discretion. All study investigators will be thoroughly trained for data management according to the Guidelines of Good Clinical Practice and considering detailed working instructions. Additionally, data entry will be cross-checked by a second study investigator and range checks for data values will be preprogrammed for data entry in the eCRF.

For quality assurance the sponsor, the Ethics Committee or an independent trial monitor may visit the research sites. Direct access to the source data and all study related files is granted on such occasions. All involved parties keep the participant data strictly confidential.

The protocol of the study was approved by the Ethics Committee of Zurich and Ethics Committee of Eastern Switzerland (EK-2022-00386). The study will be conducted according to the study protocol, the Declaration of Helsinki, the principles of Good Clinical Practice, and the Human Research Act.

How will data access and security be managed?

All research data will always be stored twice/double at the investigational sites (Institute of Human Movement Science and Sports, ETH Zurich or Department of Health, Eastern Switzerland University of Applied Sciences)

on a secure network folder located at the investigational sites and managed by the respective IT department. All data will be backed-up on a regular basis according to the regulations of the IT department at the respective investigational sites. All personal data will be pseudonymized. The correspondence table will be encrypted, and access restricted to the project leader. Trial and participant data will be handled with uttermost discretion and will only be accessible to authorized personnel who require the data to fulfil their duties within the scope of the study. The list of authorized personnel will be managed by the project leader.

How will you handle copyright and Intellectual Property Rights issues?

Copyright and intellectual property rights owners of the data generated in this project will be the ETH Zurich. We will follow the Swiss Academies of Art and Sciences regulations (2008) on scientific integrity throughout the project.

3. Data storage and preservation

How will your data be stored and backed-up during the research?

Data of all clinical outcome measures as well as the demographic and clinical characteristics of the study participants will primarily be managed and stored in the eCRF using the data management system Castor EDC (Ciwit BV, Amsterdam, The Netherlands) [3]. Separate source document of all outcomes and all other study-related data will always be stored twice/double at the investigational sites (Institute of Human Movement Science and Sports, ETH Zurich or Department of Health, Eastern Switzerland University of Applied Sciences) on a secure network folder located at the investigational sites and managed by the respective IT department. All data will be backed-up on a regular basis according to the regulations of the IT department at the respective investigational sites. Data volume is not expected to impose storage capacity at ETH Zurich and Eastern Switzerland University of Applied Sciences.

What is your data preservation plan?

All data generated in the project will be stored in several formats used in the research community, including csv.files, txt.files, R script files, R markdown files and PDF files. Using these recommended formats allow long term preservation of the data and its re-use. All data will be stored at the investigational sites (Institute of Human Movement Science and Sports, ETH Zurich or Department of Health, Eastern Switzerland University of Applied Sciences) for a minimum of ten years. After that, access to the data will only be possible under the respective terms of usage upon request.

4. Data sharing and re-use

How and where will the data be shared?

Datasets from this project which underpin a publication will be deposited in the Zenodo repository and made public after completing data collection. Data in the repository will be stored in accordance with funder's data

policies. Files deposited in the Zenodo repository will be given a Digital Object Identifier (DOI). The retention schedule for data will be set to 10 years from date of deposition in the first instance, with possible extension for datasets which remain in regular use.

The DOI issued to datasets in the repository can be included as part of a data citation in publications, allowing the datasets underpinning a publication to be identified and accessed.

Metadata about datasets held in the Zenodo repository will be publicly searchable and discoverable and will indicate how and on what terms the dataset can be accessed.

Are there any necessary limitations to protect sensitive data?

Access to the data will be granted to third parties at the time of the scientific publication of the results. Health-related data are sensitive personal data by the Swiss Federal Act on Data Protection. The data can only be used for research purposes and sharing with third parties upon the consent of the participants and after ethical approval. Only anonymized data will be shared.

All digital repositories I will choose are conform to the FAIR Data Principles

YES, we will choose digital repositories that are conform to the FAIR Data Principles.

I will choose digital repositories maintained by a non-profit organisation

YES, we will choose digital repositories maintained by a non-profit organization.

5. References

1. Chan, A.W., et al., *SPIRIT 2013 explanation and elaboration: guidance for protocols of clinical trials.* Bmj-British Medical Journal, 2013. ***346***: p. e7586.
2. Chan, A.W., et al., *SPIRIT 2013 Statement: Defining Standard Protocol Items for Clinical Trials.* Annals of Internal Medicine, 2013. ***158***(3): p. 200-+.
3. Castor, E.D.C. *Castor Electronic Data Capture*. 2019 August 28, 2019]; Available from: https://castoredc.com.
